# Supplementary material for: Discovery, characterization, and comparative analysis of new UGT72 and UGT84 family glycosyltransferases
Source: Commun Chem. 2024 Jun 28;7:147. doi: 10.1038/s42004-024-01231-1 (PMC11213884; doi:10.1038/s42004-024-01231-1)
Supplement: Supplementary file 3 — Description of Additional Supplementary Files [file 42004_2024_1231_MOESM3_ESM.pdf]

Description of Additional Supplementary Files

File name- Supplementary Data 1

File description- Supplementary file containing the source data for Figures 3, 4 and 5 of the paper
