# Supplementary material for: Discovery, characterization, and comparative analysis of new UGT72 and UGT84 family glycosyltransferases
Source: Commun Chem. 2024 Jun 28;7:147. doi: 10.1038/s42004-024-01231-1 (PMC11213884; doi:10.1038/s42004-024-01231-1)
Supplement: Supplementary file 4 — Supplementary Data 1 [file 42004_2024_1231_MOESM4_ESM.docx]

**Supplementary Information**

**Discovery, characterization, and comparative analysis of new UGT72 and UGT84 family glycosyltransferases**

Tuo Li^1*^, Annika J. E. Borg^1*^, Leo Krammer^2^, Hansjörg Weber^2^, Rolf Breinbauer^2^ & Bernd Nidetzky^1,3**^

^1^Institute of Biotechnology and Biochemical Engineering, Graz University of Technology, NAWI Graz, Petersgasse 12/1, 8010 Graz, Austria

^2^Institute of Organic Chemistry, Graz University of Technology, NAWI Graz, Stremayrgasse 9, 8010 Graz, Austria

^3^Austrian Centre of Industrial Biotechnology (acib), Krenngasse 37, 8010 Graz, Austria

^*^These authors contributed equally.

^**^Corresponding author: Bernd Nidetzky (bernd.nidetzky@tugraz.at)

**Supplementary Methods**

**Materials**

Sinapic acid (>98%), coniferyl aldehyde (>98%), *p*-HAP (>99%), picein (>98%), apigenin 7-*O*-glucoside (>97%), luteolin 7-*O*-glucoside (>98%), UDP-galactose (>95%), and UDP-glucuronic acid (>98%) were from Sigma Aldrich (Vienna, Austria). Phlorizin (>98%) was from Roth (Karlsruhe, Germany). Phloretin (>98%), apigenin (>97%), luteolin (>98%), daidzein (>98%), daidzin (>97%), UDP (>97%) and UDP-glucose (>98%) were from Carbosynth (Compton, UK). UDP-xylose (>99%) was obtained from a previous study using enzymatic synthesis^1^. All other reagents and chemicals were of highest available purity and purchased from Sigma Aldrich/Fluka (Vienna, Austria), Roth (Karlsruhe, Germany) or Merck (Vienna, Austria).

**Enzyme production**

The cells harboring pET-28a_UGT (UGT72D1, UGT72D7, UGT84A49, UGT84A119) were cultivated individually in 10 mL of Terrific Broth (TB) medium with 50 µg mL^-1^ kanamycin at 37 °C for 16 h. From the pre-culture, 2 mL were used to inoculate fresh TB medium (250 mL) supplemented with 2% (v/v) glycerol and 50 µg mL^-1^ kanamycin, and the cells were grown at 37 °C and 120 rpm. Upon reaching a cell density (OD_600_) of 1.0, gene expression was induced by adding isopropyl β-d-thiogalactoside (IPTG; final concentration of 0.2 mM). The induced cells were incubated at 18 °C and 120 rpm for 20 h. The cells were harvested by centrifugation (3010 g, 4 °C, 30 min), followed by re-suspending the pellet in 10 mL of His-tag loading buffer (20 mM Na_2_HPO_4_, 500 mM NaCl, 20 mM imidazole, pH 7.4). The cell suspension was stored overnight at -20 °C prior to cell lysis. Sonication (Sonic Dismembrator Model 505, Fisher Scientific, Vienna, Austria) was employed for cell disruption (pulse 4 sec on, 6 sec off, 60% amplitude, 6 min), and the lysate was centrifuged at 4 °C, 21130 g for 60 min. The resulting supernatant, filtered through a 0.45 µm filter, was loaded onto the HisTrap^TM^ HP column (5 mL resin, Cytiva, Uppsala, Sweden) pre-equilibrated with the loading buffer. The column was washed with 50 mL loading buffer at the flowrate of 2 mL min^-1^. The elution of target protein was achieved by applying a gradient of 0-100% elution buffer (20 mM Na_2_HPO_4_, 500 mM NaCl, 250 mM imidazole, pH 7.4) over 60 min. The pure fractions containing the target protein were pooled and subjected to buffer exchange against 50 mM Tris-HCl buffer (pH 7.4) supplemented with 1.0 mM DTT and 10% (v/v) glycerol using Vivaspin 20 ultrafiltration tubes (30,000 MWCO PES; Sartorius, Goettingen, Germany). After buffer exchange, the protein was divided into aliquots, flash frozen in liquid nitrogen, and stored at -20 °C. Protein concentration was determined based on the absorption at 280 nm on a spectrophotometer (Nanophotometer N50, IMPLEN, Munich, Germany) using the molar extinction coefficient (Supplementary Table 1) calculated from the amino acid sequence.

**Enzyme activity assays**

Activity assays toward sinapic acid and coniferyl aldehyde

The reaction mixture (100 µL) contained 1.0 mM sinapic acid or coniferyl aldehyde (dissolved in DMSO at a final concentration of 2%), 2.0 mM UDP-Glc and 0.0050-0.50 mg mL^-1^ UGT in 50 mM potassium phosphate buffer. For activity assays toward sinapic acid, UGT84A119/UGT84A49 (0.005 mg mL^-1^) were employed at pH 5.0, while 0.05 mg mL^-1^ UGT72D7 or 0.5 mg mL^-1^ UGT72D1 were used at pH 8.0. For activity assays toward coniferyl aldehyde, UGT72D1/UGT72D7 (0.005 mg mL^-1^) or UGT84A49/UGT84A119 (0.5 mg mL^-1^) were utilized at pH 8.0. The reactions were incubated at 30 °C without agitation, quenched with methanol (75% (v/v) final concentration) at desired time points, and the precipitated enzymes removed by centrifugation (21130 g, 4 °C, 30 min) prior to HPLC analysis. The initial formation rates for products (glucosides or glucose ester) were determined from the corresponding linear parts of the time courses, by dividing the slope of the linear regression (mM min^-1^) by the enzyme concentration (mg mL^-1^), providing the initial rate in µmol (min mg protein) ^-1^. One unit (U) of UGT activity is defined as the amount of enzyme producing 1 µmol of product (*O*-glucoside or glucose ester) per minute under conditions used, where the acceptor substrate is present in excess. All the activity assays were performed in duplicates (N=2).

Activity assays toward selected phenolic substrates

The reaction mixture (100 µL) contained 1.0 mM acceptor substrate (*p*-HAP, phloretin, apigenin, luteolin or daidzein; dissolved in DMSO at a final concentration of 2%), 2.0 mM UDP-sugar (UDP-glucose, UDP-xylose, UDP-galactose, or UDP-glucuronic acid), and 0.5 mg mL^-1^ UGT (UGT84A119, UGT84A49, UGT72D1, UGT72D7) in potassium phosphate buffer (50 mM, pH 8.0). The reactions were conducted and analyzed as described under “Activity assays toward sinapic acid and coniferyl aldehyde”.

**Preparation and isolation of products**

Sinapic acid 4-*O*-glucoside

The reaction (50 mL) was carried out in a 250 mL flask under the following conditions: 2.0 mM sinapic acid (dissolved in DMSO at a final concentration of 2%), 4.0 mM UDP-Glc, 0.10 mg mL^-1^ UGT72D1, in potassium phosphate buffer (50 mM, pH 8.0). The reaction was performed at 30 °C, and stirred at 500 rpm. After 24 h, 10 µL of reaction mixture were sampled as described under “Activity assay toward sinapic acid and coniferyl aldehyde”, and subjected to HPLC analysis (conversion rate of sinapic acid 4-*O*-glucoside, 53%). The reaction was quenched by removing the enzymes using Vivaspin 20 ultrafiltration tubes (10,000 MWCO PES; Sartorius, Goettingen, Germany), at 3220 g and 4 °C. Subsequently, solvent in the reaction mixture was removed under reduced pressure on a Laborota 4000 rotary evaporator (Heidolph, Schwabach, Germany) at 40 °C. The crude product was resuspended in 2.0 mL solvent mixture of 1-butanol and methanol (4:1), and loaded into a silica 60 column (30 mL, 0.04-0.063 mm, Machery-nagel, Duren, Germany) for purification of sinapic acid 4-*O*-glucoside. The column was washed with the solvent mixture, and the eluted fractions analyzed on HPLC. The product-containing fractions were pooled and the solvent removed by rotary evaporator (as described above) and lyophilization (Christ Alpha 1-4 lyophilizer, B. Braun Biotech International, Melsungen, Germany).

Sinapic acid glucose ester

The reaction was performed as described under “Sinapic acid 4-*O*-glucoside”, except that 0.10 mg mL^-1^ UGT84A119 was used in potassium phosphate buffer (50 mM, pH 5.0). After 24 h, the reaction was sampled and analyzed on HPLC (conversion rate of sinapic acid glucose ester, 35%). The target product was isolated as described under “Sinapic acid 4-*O*-glucoside”.

Coniferyl aldehyde 4-*O*-glucoside

The reaction was performed as described under “Sinapic acid 4-*O*-glucoside”, except that 2.0 mM coniferyl aldehyde (dissolved in DMSO at a final concentration of 2%) was used. After 24 h, the reaction was sampled and analyzed on HPLC (conversion rate of coniferyl aldehyde 4-*O*-glucoside, 95%). Afterwards, the enzymes and the solvent were removed as described under “Sinapic acid 4-*O*-glucoside”, the crude product redissolved in 2 mL methanol and loaded onto silica plates (20 cm × 20 cm, layer thickness 2 mm; Merck, Darmstadt, Germany). The thin-layer chromatography (TLC) was performed with eluent 1-butanol/acetic acid/deionized water (2:1:1) and the area corresponding to coniferyl aldehyde 4-*O*-glucoside located under a UV lamp (Supplementary Figure 37). The target product was scratched off the plate with a spatula, and extracted from the silica powder with 15 mL methanol in 50 mL Sarstedt tube by manual inversion every 15 min for 1 h. Subsequently, the solvent was removed by rotary evaporator and lyophilization (as described above).

Phloretin 4′-*O*-glucoside

The reactions (15 × 1.0 mL) were carried out in 2 mL Eppendorf tubes under the following reaction conditions: 2.0 mM phloretin (dissolved in DMSO at a final concentration of 4%), 4.0 mM UDP-Glc, 5.0 mg mL^-1^ UGT84A119, in potassium phosphate buffer (50 mM, pH 8.0). The reactions were conducted at 30 °C without agitation. After 24 h, additional UGT84A119 (3.0 mg mL^-1^) was added into the reaction mixture. After 30 h, the reaction was sampled and analyzed on HPLC (conversion rate of phloretin 2′-*O*-glucoside and 4′-*O*-glucoside, 7% and 63%, respectively). The reactions were quenched by removing the enzymes as described under “Sinapic acid 4-*O*-glucoside”. The filtered mixture was lyophilized, re-dissolved in deionized water and loaded into a column containing silica gel C18 (20 mL, 0.025-0.07 mm, ROTH, Karlsruhe, Germany). Deionized water (30 mL) and a mixture of acetonitrile/deionized water (1:9; 50 mL) was used to wash out impurities and phloretin 4′-*O*-glucoside was eluted with 50 mL of acetonitrile/deionized water (1:4) mixture. The fractions (5 mL) were analyzed on TLC and by HPLC-MS. The pure fractions containing the target product were lyophilized to give the pure product.

Apigenin 4′-*O*-glucoside & apigenin 7,4′-di-*O*-glucoside

The reactions (20 × 1.0 mL) were carried out in 2 mL Eppendorf tubes under the following reaction conditions: 2.0 mM apigenin (dissolved in DMSO at a final concentration of 10%), 6.0 mM UDP-Glc, 0.50 mg mL^-1^ UGT84A49, in potassium phosphate buffer (50 mM, pH 8.0). Reactions were conducted at 30 °C without agitation. After 24 h, the reaction was sampled and analyzed by HPLC (conversion rate of apigenin 7-*O*, 4′-*O*, and 7,4′-di-*O*-glucoside, 16%, 15% and 35%, respectively). The reactions were quenched, and lyophilized as described under “Phloretin 4′-*O*-glucoside”. The product mixture was re-dissolved in a 1:1 mixture of deionized water and DMSO, and subjected to preparative HPLC for product isolation using the method “*Method Prep_1*” as described under “Reversed-Phase Preparative HPLC”.

Luteolin 3′-*O*-glucoside

The reactions were performed as described under “Phloretin 4′-*O*-glucoside”, except that 2.0 mM luteolin (dissolved in DMSO at a final concentration of 10%), and 0.50 mg mL^-1^ UGT72D1 was used. The reactions were quenched at 24 h (conversion rate of luteolin 7-*O* and 3′-*O*-glucoside, 35% and 63%, respectively). The enzyme was removed and the supernatant lyophilized as described under “Phloretin 4′-*O*-glucoside”. The product mixture was re-dissolved in DMSO, and subjected to preparative HPLC for product isolation using the method “*Method Prep_2*” as described under “Reversed-Phase Preparative HPLC”.

Luteolin 4′-*O*-glucoside & luteolin 7,4′-di-*O*-glucoside

The reactions were performed as described under “Apigenin 4′-*O*-glucoside”, except that 2.0 mM luteolin (dissolved in DMSO at a final concentration of 10%) was used. After 24 h, the reaction was sampled and analyzed by HPLC (conversion rate of luteolin 7-*O*, 4′-*O*, and 7,4′-di-*O*-glucoside, 23% 22%, and 45%, respectively). The enzyme removal and supernatant lyophilization were performed as described under “Phloretin 4′-*O*-glucoside”. The product mixture was re-dissolved in a mixture of deionized water and DMSO (1:1), and the individual products isolated as described under “Luteolin 3′-*O*-glucoside”.

**Analytical methods**

HPLC-UV/MS

Analytical HPLC-UVMS measurements were performed on an Agilent Technologies 1200 Series system (G1379 Degasser, G1312 Binary Pump, G1367C HiP ALS SL Autosampler, G1330B FC/ALS Thermostat, G1316B TCC SL column compartment, G1365C MWD SL multiple wavelength detector (deuterium lamp, 190-400 nm)) equipped with a single quadrupole LCMS detector “6120 LC/MS” using electrospray ionization source (ESI in positive and negative mode). Separations were carried out on a C-18-Reversed-Phase column of the type “Poroshell^®^ 120 SB-C18, 3.0 × 100 mm, 2.7 μm” by Agilent Technologies. Flow: Constant flow rate 0.7 mL min^-1^, T = 35 °C. The following method was used: *MeCN_2_100*: 0.0-0.1 min, isocratic, 2% MeCN (98% H_2_O + 0.05% TFA); 0.1- 8.0 min, linear, 2% to 100% MeCN (98% to 0% H_2_O + 0.05% TFA); 8.0-11.1 min, isocratic, 100% MeCN; 11.1-11.3 min, linear, 100% to 2% MeCN (0% to 98% H_2_O + 0.05 % TFA); 11.3-12.0 min, isocratic, 2% MeCN (98% H_2_O + 0.05% TFA).

Reversed-Phase Preparative HPLC

Reversed phase preparative HPLC purifications were performed on a Thermo Scientific UltiMate 3000 system. Detection was accomplished with a Dionex UltiMate Diode Array Detector. The separations were carried out on a Macherey Nagel 125/21 Nucleodur® 100-5 C18EC (125 × 21 mm, 5 µm) column. Acetonitrile and water with 0.05%-0.1% trifluoroacetic acid were used as eluents for the purification of the compounds. Typically, the product fractions obtained from preparative HPLC were concentrated under reduced pressure to remove the organic solvents and then lyophilized to obtain the product.

The following methods were applied:

*Method Prep_1***:** T = 30 °C, constant flow rate: 15 mL min^-1^; 0.0-3.0 min, isocratic, 2% MeCN (98% H_2_O + 0.1% TFA), 3.0-16.0 min, linear, 2% to 50% MeCN (98% to 50% H_2_O + 0.1% TFA), 16.0-17.0 min, linear, 50% to 100% MeCN (50% to 0% H_2_O + 0.1% TFA), 17.0-18.0 min, isocratic, 100% MeCN, 18.0-19.0 min, linear, 100% to 2% MeCN (0% to 98% H_2_O + 0.1% TFA), 19.0-22.0 min, isocratic, 2% MeCN (98% H_2_O + 0.1% TFA).

*Method Prep_2:* T = 30 °C, constant flow rate: 15 mL min^-1^; 0.0-3.0 min, isocratic, 2% MeCN (98% H_2_O + 0.05% TFA), 3.0-13.0 min, linear, 2% to 50% MeCN (98% to 50% H_2_O + 0.05% TFA), 13.0-14.0 min, linear, 50% to 100% MeCN (50% to 0% H_2_O + 0.05% TFA), 14.0-15.0 min, isocratic, 100% MeCN, 15.0-16.0 min, linear, 100% to 2% MeCN (0% to 98% H_2_O + 0.05% TFA), 16.0-19.0 min, isocratic, 2% MeCN (98% H_2_O + 0.05% TFA).

**Supplementary Table 1**. Basic information of chosen UGT72 and UGT84 candidates.

| Entry | Abbreviation | Organism | Extinction coefficient  (M^-1^ cm^-1^) |
| --- | --- | --- | --- |
| 1 | UGT84A49 | *Fragaria ananassa* | 61015 |
| 2 | J045 | *Brassica napus* | 53565 |
| 3 | UGT84A119 | *Fagus sylvatica* | 68005 |
| 4 | UGT72D1 | *Arabidopsis thaliana* | 70275 |
| 5 | UGT72D7 | *Citrus sinensis* | 59025 |
| 6 | KZ95 | *Artemisia annua* | 52745 |

**Supplementary Table 2**. Amino acid sequence identities between the putative UGT proteins and templates.

|  | UGT84A49 | UGT84A119 | J045 | UGT72D1 | UGT72D7 | KZ95 |
| --- | --- | --- | --- | --- | --- | --- |
| UGT72E3 | 26 | 26 | 22 | 41 | 48 | 44 |
| UGT84A2 | 53 | 56 | 57 | 24 | 24 | 25 |

**Supplementary Table 3**. GenBank accession numbers of sequences used in the phylogenetic analysis.

| **UGT** | **Access number** | **UGT** | **Access number** | **UGT** | **Access number** |
| --- | --- | --- | --- | --- | --- |
| UGT71A15 | DQ103712 | UGT71A16 | FJ854494 | UGT71K1 | FJ854493 |
| UGT71K2 | FJ854495 | UGT71S4 | QDM38908.1 | UGT72AF1 | AOG18239.1 |
| UGT72AM1 | ASA40331.1 | *Ht*UGT72AS1 | UXB92752.1 | *Nb*UGT72AY1 | UHH90560.1 |
| *St*UGT72AY2 | XP_015164078.1 | UGT72AZ2 | QLI54351.1 | UGT72B1 | OAP00532.1 |
| UGT72B3 | NP_001322773.1 | UGT72B11 | ACB56923.1 | UGT72B14 | ANS59121.1 |
| UGT72B37 | QLI54353.1 | UGT72B39 | QLI54355.1 | UGT72B42 | QDM38905.1 |
| UGT72BD1 | QHB92369.1 | UGT72C1 | NP_195395.4 | UGT72D1 | Q9ZU72.1 |
| UGT72E1 | NP_566938.1 | UGT72E2 | NP_201470.1 | UGT72E3 | NP_198003.1 |
| UGT72E8 | UTO68652.1 | UGT72V3 | AOG18244.1 | UGT72X4 | XP_003532193.1 |
| UGT72X5 | QDM38910.1 | UGT72Z2 | AKK25344.1 | UGT72Z3 | XP_003532192.3 |
| *Pa*GT2 | BAG71125.1 | UGT73A10 | BAG80536.1 | UGT73B1 | NP_567955.1 |
| UGT73B28 | QDM38901.1 | UGT73C1 | NP_181213.1 | UGT73C6 | NP_181217.1 |
| UGT73P12 | BBN60799.1 | UGT74B1 | NP_173820.1 | UGT74D1 | OAP11252.1 |
| UGT74F1 | NP_181912.1 | UGT74F2 | NP_181910.1 | UGT74G1 | AAR06920.1 |
| UGT74M1 | ABK76266.1 | UGT75B1 | OAP16927.1 | UGT75L17 | AY786997 |
| UGT75L25 | QCC89365.1 | UGT75X1 | QCC89364.1 | UGT76C1 | NP_196206.1 |
| UGT76C2 | NP_196205.1 | UGT76E1 | NP_200766.2 | UGT76G1 | AAR06912.1 |
| UGT78D1 | NP_564357.1 | UGT78D3 | NP_197205.1 | UGT78K6 | BAF49297.1 |
| UGT79A6 | NP_001275524.1 | UGT84A1 | NP_193283.2 | UGT84A2 | NP_188793.1 |
| UGT84A3 | NP_193284.1 | UGT84A9a | CAS03346.1 | UGT84A9c | CAS03348.1 |
| UGT84A13 | AHA54051.1 | *Pg*UGT84A23 | ANN02875.1 | UGT84A25a | BBB21213.1 |
| UGT84A26a | BBB21215.1 | UGT84A33 | AYW01718.1 | UGT84A34 | BBA68563.1 |
| UGT84A57 | BBI55602.1 | UGT84A68 | WFR85803.1 | UGT84A77 | QZM06937.1 |
| UGT84B1 | NP_179907.1 | UGT84F6 | QDM38904.1 | UGT84F9 | XP_013470035.1 |
| *Fa*GT2 | AAU09443.1 | SGT1 | AAF98390.1 | *Vv*gGT1 | AEW31187.1 |
| *Vv*gGT2 | AEW31188.1 | *Vv*gGT3 | NP_001267849.1 | VLRSgt | ABH03018.1 |
| UGT85A19 | ABV68925.1 | UGT85C2 | AAR06916.1 | UGT85K11 | BAO51834.1 |
| UGT88A29 | QDM38900.1 | UGT88A32 | MN381003 | UGT88A33 | MN381009 |
| UGT88F1 | EU246349 | UGT88F2 | FJ854496 | UGT88F4 | KX639792 |
| UGT88F6 | KC895981 | UGT88F7 | KC895982 | UGT88F8 | KC895983 |
| *Pg*UGT94B1 | AGR44632.1 | UGT94P1 | BAO51835.1 | UGT708A6 | NP_001132650.2 |
| UGT708A11 | QLF98873.1 | UGT708B4 | QGL05036.1 | UGT708C1 | BAP90360.1 |
| UGT708G1 | BBA18062.1 | UGT708G2 | BBA18063.1 | *Mp*UGT737B1 | PTQ47498.1 |
| *Vv*GT1 | NP_001384786.1 | UGT71E5 | AOC55048.1 | *Os*CGT | CAQ77160.1 |

**Supplementary Table 4**. HPLC columns and methods used in the analysis of the UGT reactions. MeCN = acetonitrile.

|  | HPLC columns | Methods |
| --- | --- | --- |
| UDP-sugar/UDP in all reactions (except for *p*-HAP reactions) | Kinetex C18 (5 µm, 100 Å, 50 × 4.6 mm) | Flow rate, 2.0 mL min^-1^; 0-2 min, isocratic, 5% MeCN. |
| UDP-sugar/UDP in *p*-HAP reactions | Kinetex C18 (5 µm, 100 Å, 150 × 4.6 mm) | Flow rate, 0.8 mL min^-1^; 0-6 min, isocratic, 20% MeCN. |
| Acceptor/product in all reactions (except for apigenin reactions) | Kinetex C18 (5 µm, 100 Å, 150 × 4.6 mm) | Flow rate, 1.0 mL min^-1^; 0-5.5 min, linear, 20% to 75% MeCN; 5.5-7.5 min, isocratic, 75% MeCN; 7.5-7.51 min, linear, 75% to 20% MeCN; 7.51-9 min, isocratic, 20% MeCN. |
| Acceptor/product in apigenin reactions | Kinetex C18 (3 µm, 100 Å, 200 × 4.6 mm) | Flow rate, 0.6 mL min^-1^; 0-15 min, linear, 20% to 75% MeCN; 15-16 min, isocratic, 75% MeCN; 16-16.01 min, linear, 75% to 20% MeCN; 16.01-18 min, isocratic, 20% MeCN. |


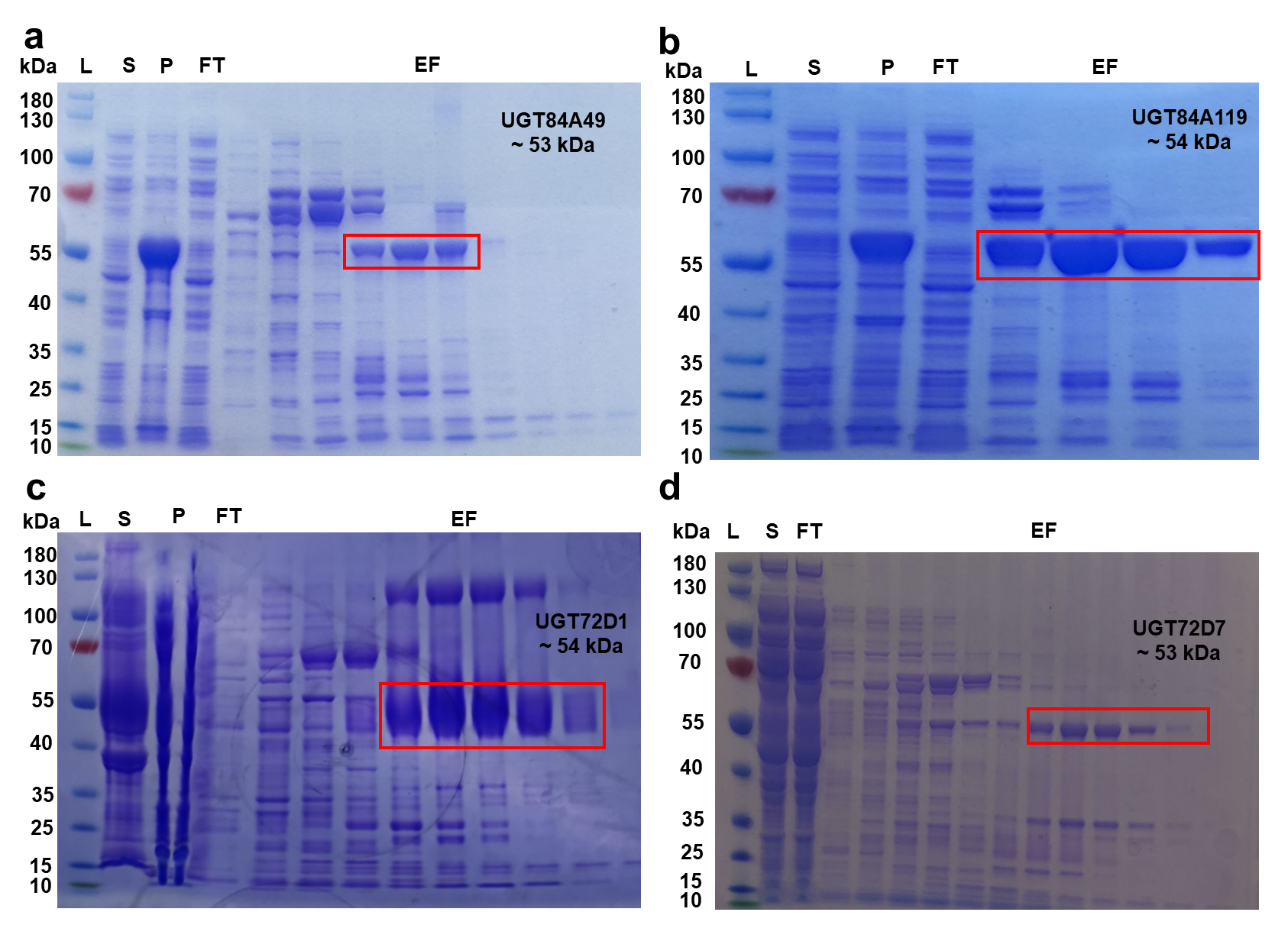


**Supplementary Figure 1**. SDS-gels from the His-tag purification of UGT84A49 (**a**), UGT84A119 (**b**), UGT72D1 (**c**) and UGT72D7 (**d**). L, molecular mass marker; S, supernatant; P, pellet; FT, flow through (unbound proteins); EF, elution fractions.

**
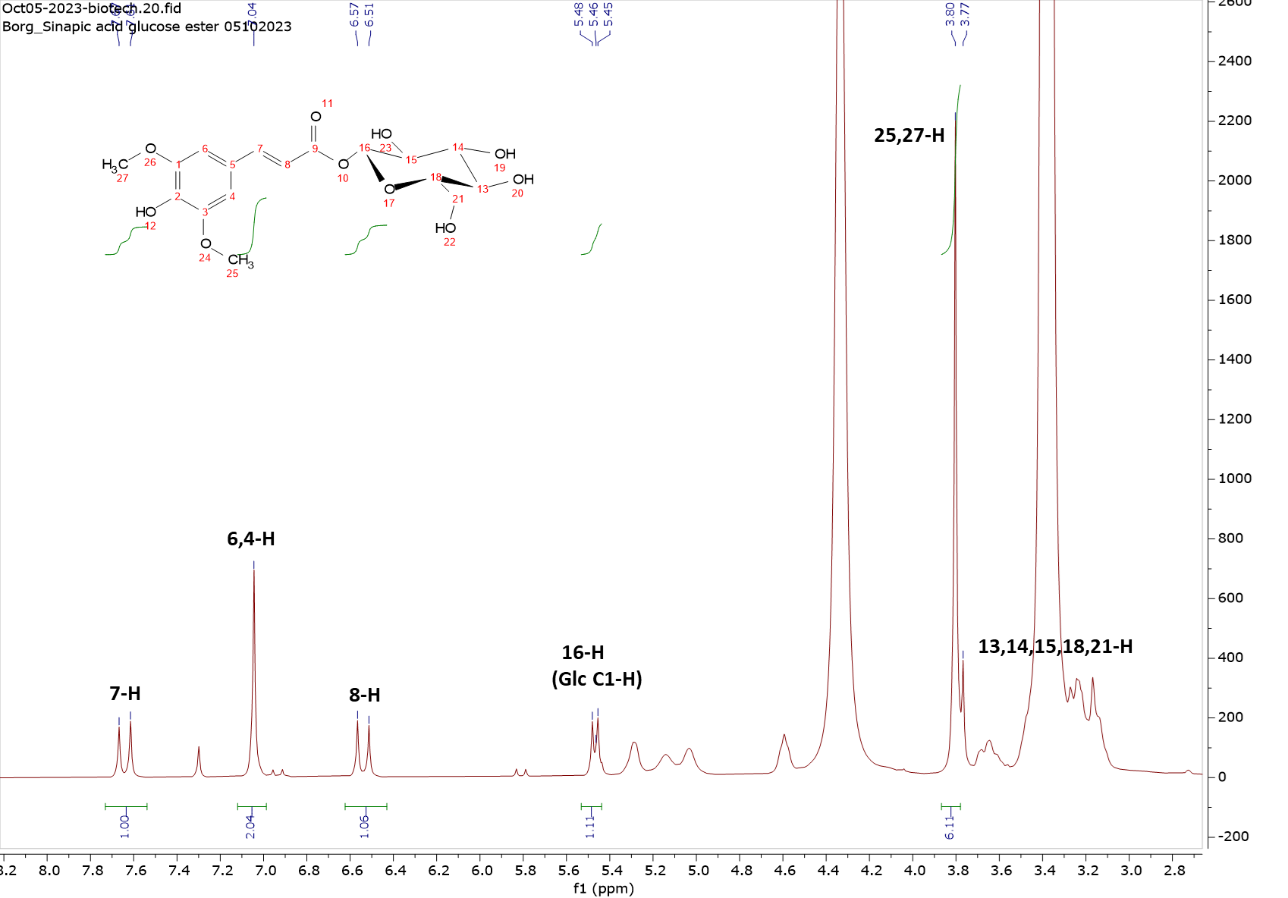
**

**Supplementary Figure 2**. ^1^H NMR spectrum (300 MHz, D_2_O) of isolated sinapic acid glucose ester: δ 7.65 ppm (d, 1H), 7.04 ppm (s, 2H), 6.54 ppm (d, 1H), 5.46 ppm (d, 1H), 3.78 ppm (s, 6H). The signals from glucose C2-OH, C3-OH, C4-OH, C5-OH and C6-OH are under the signal of methanol at 3.34 ppm.

**
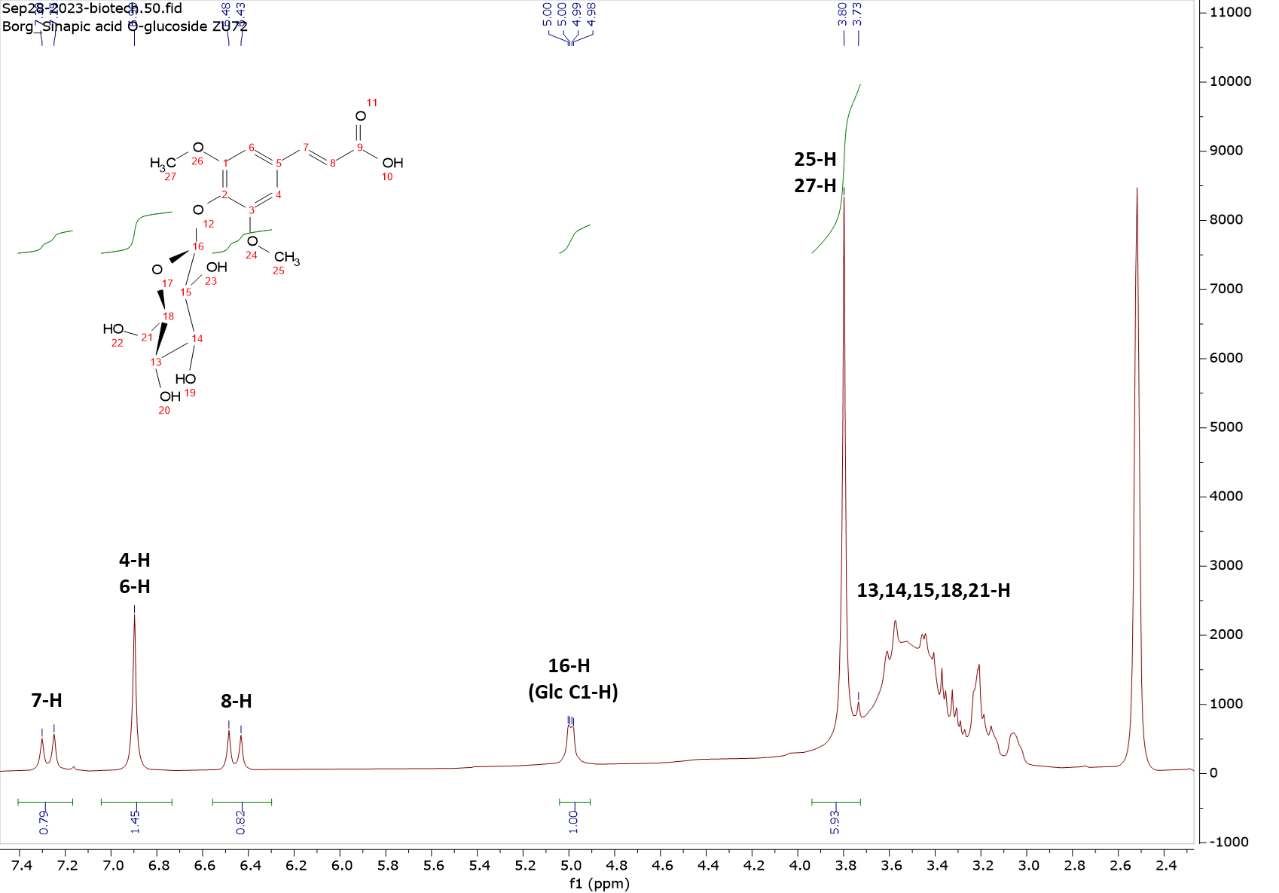
**

**Supplementary Figure 3**. ^1^H NMR spectrum (300 MHz, D_2_O) of isolated sinapic acid 4-*O*-glucoside: δ 7.30 ppm (d, 1H), 6.90 ppm (s, 2H), 6.45 ppm (d, 1H), 4.99 ppm (d, 1H), 3.76 ppm (s, 6H). The signals from glucose C2-OH, C3-OH, C4-OH, C5-OH and C6-OH appear together at 3.2-3.6 ppm.

**
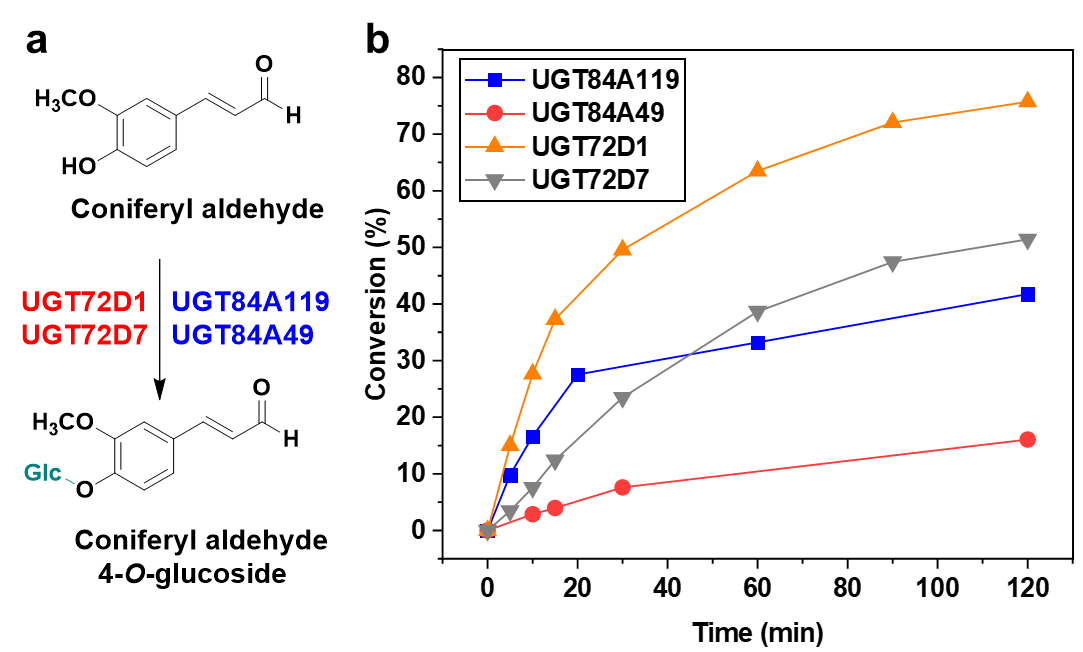
**

**Supplementary Figure 4**. Reaction scheme and time courses for UGT reactions toward coniferyl aldehyde. **a**. Reaction scheme from coniferyl aldehyde to corresponding 4-*O*-glucoside catalyzed by UGT72D1, UGT72D7, UGT84A119, or UGT84A49. **b**. Time course of product formation in UGT reactions for measuring the enzymatic activities. Reactions (100 μL) contained 1.0 mM coniferyl aldehyde, 2.0 mM UDP-Glc and 0.0050-0.50 mg mL^-1^ enzymes in 50 mM potassium phosphate buffer (pH 8.0).

**
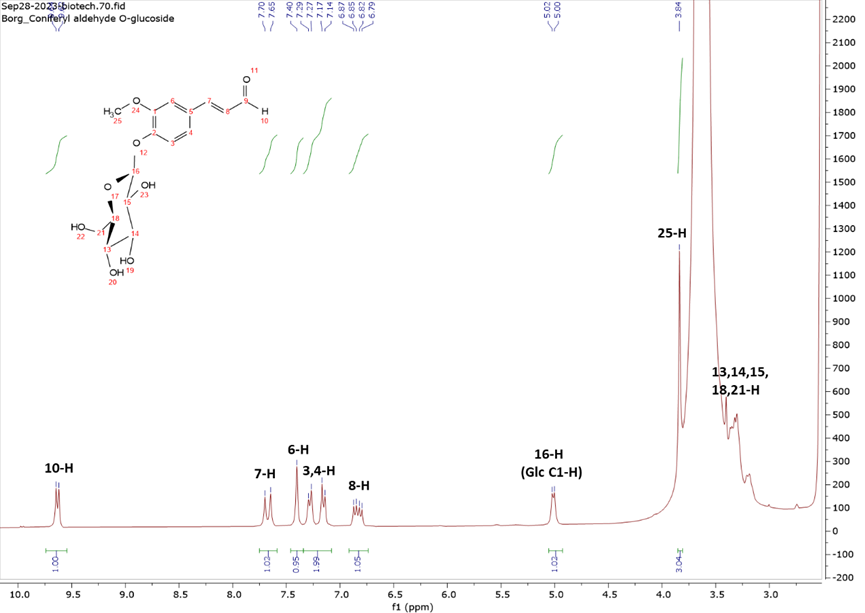
**

**Supplementary Figure 5**. ^1^H NMR spectrum (300 MHz, D_2_O) of isolated coniferyl aldehyde 4-*O*-glucoside: δ 9.65 ppm (d, 1H), 7.67 ppm (d, 1H), 7.40 ppm (s, 1H), 7.27 ppm (dd, 2H), 6.85 ppm (dd, 1H), 5.01 ppm (d, 1H), 3.84 ppm (s, 3H). The signals from glucose C2-OH, C3-OH, C4-OH, C5-OH and C6-OH appear together at 3.2-3.6 ppm.


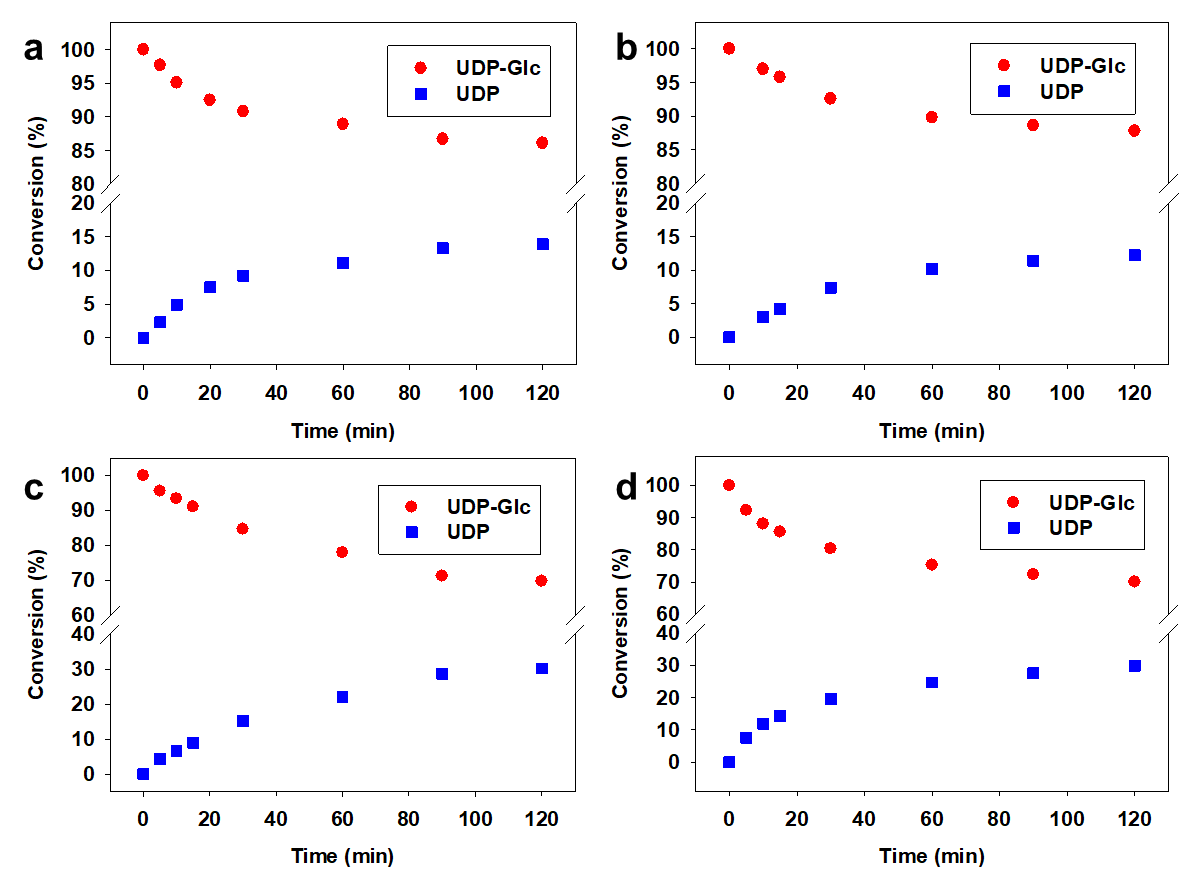


**Supplementary Figure 6**. Time course of UDP/UDP-Glc in UGT84A119 (**a**), UGT84A49 (**b**), UGT72D7 (**c**) and UGT72D1 (**d**) reactions toward sinapic acid. Reactions (100 μL) contained 1.0 mM sinapic acid, 2.0 mM UDP-Glc and 0.0050-0.50 mg mL^-1^ enzymes in 50 mM potassium phosphate buffer (pH 5.0 or 8.0).


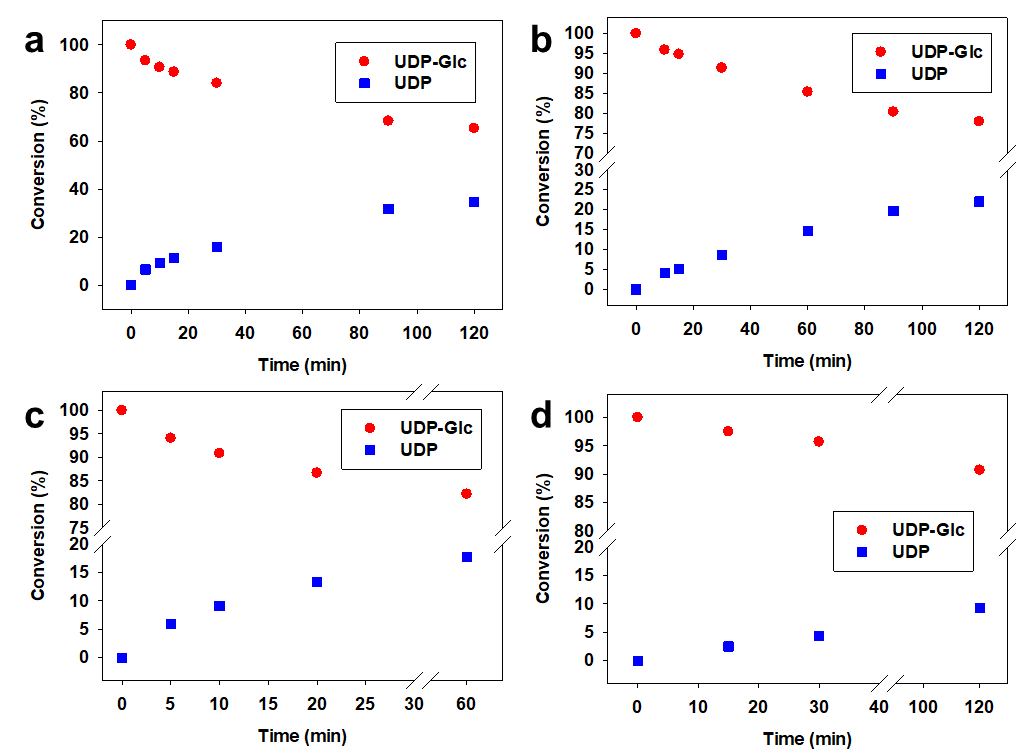


**Supplementary Figure 7**. Time course of UDP/UDP-Glc in UGT72D1 (**a**), UGT72D7 (**b**), UGT84A119 (**c**) and UGT84A49 (**d**) reactions toward coniferyl aldehyde. Reactions (100 μL) contained 1.0 mM coniferyl aldehyde, 2.0 mM UDP-Glc and 0.0050-0.50 mg mL^-1^ enzymes in potassium phosphate buffer (50 mM, pH 8.0).


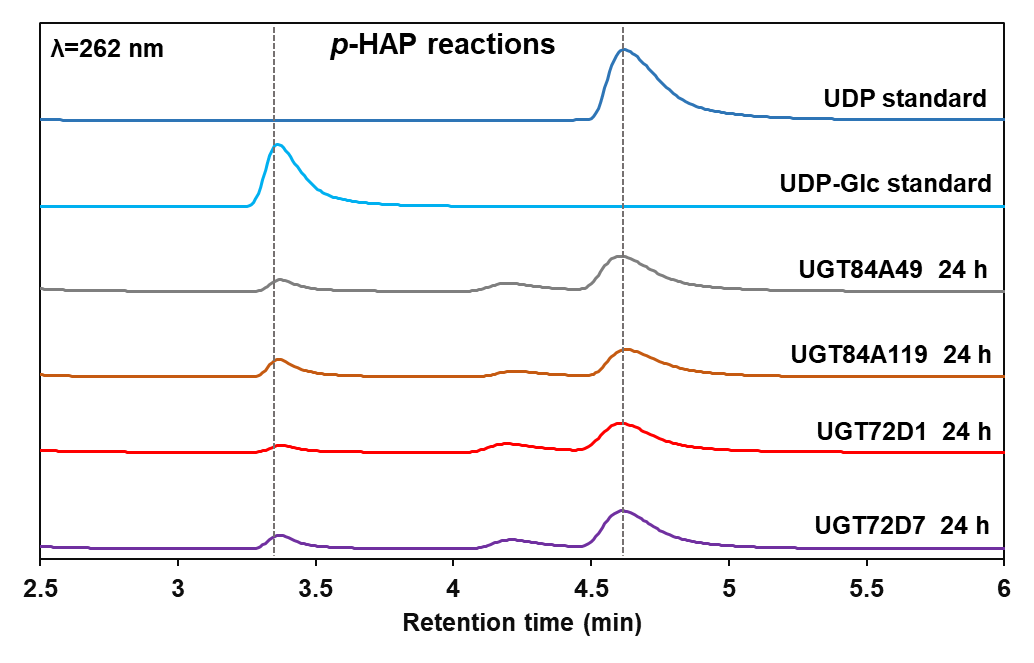


**Supplementary Figure 8**. Overlay of HPLC chromatograms for UDP/UDP-Glc in UGT reactions toward *p*-HAP. Reactions (100 μL) contained 1.0 mM *p*-HAP, 2.0 mM UDP-Glc and 0.50 mg mL^-1^ enzymes in potassium phosphate buffer (50 mM, pH 8.0).


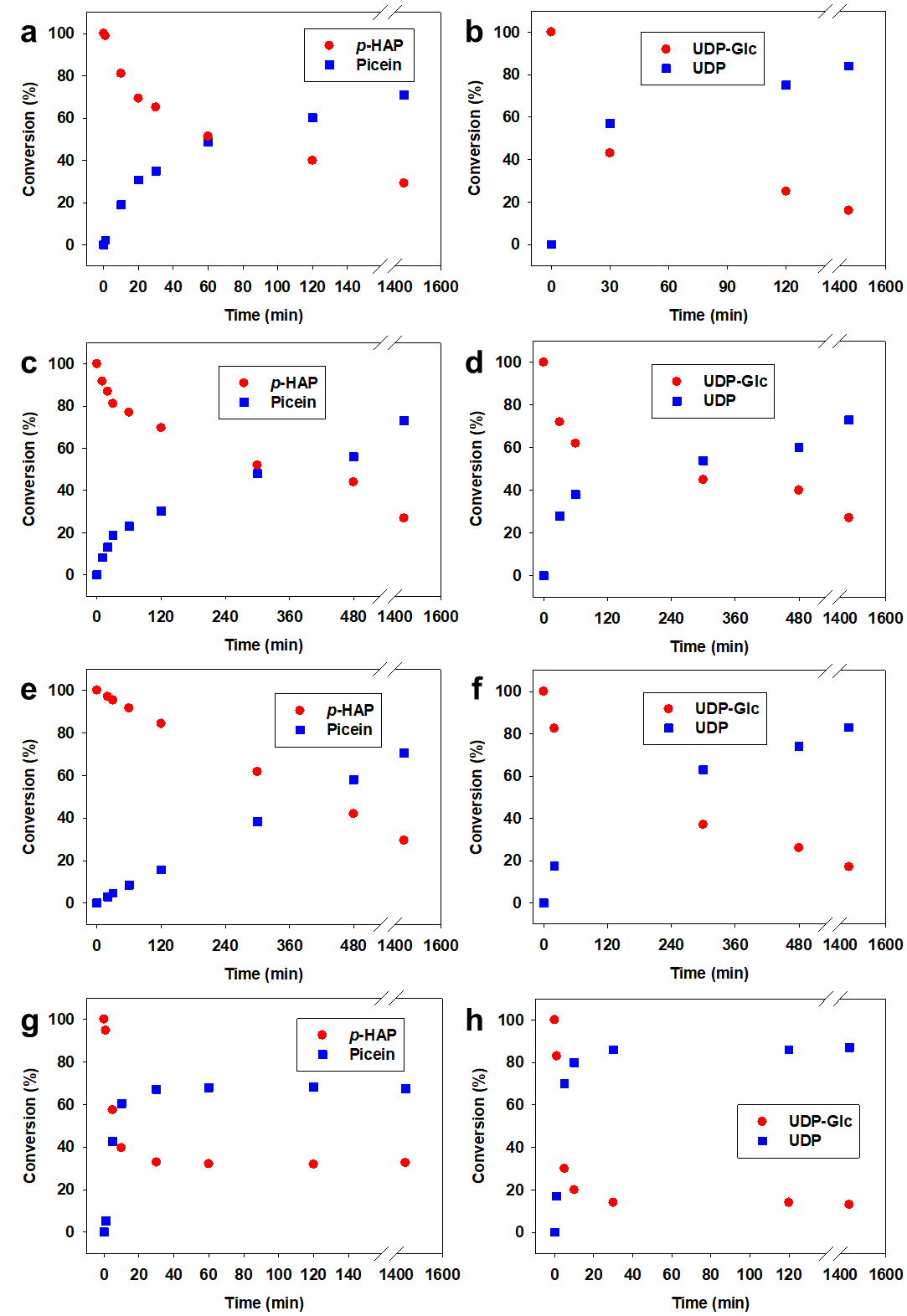


**Supplementary Figure 9**. Time courses of *p*-HAP/picein and UDP-Glc/UDP in UGT/*p*-HAP reactions: UGT84A49 (**a**, **b**), UGT84A119 (**c**, **d**), UGT72D7 (**e**, **f**) and UGT72D1 (**g**, **h**) reactions. Reactions (100 μL) contained 1.0 mM *p*-HAP, 2.0 mM UDP-Glc and 0.50 mg mL^-1^ enzymes in potassium phosphate buffer (50 mM, pH 8.0).


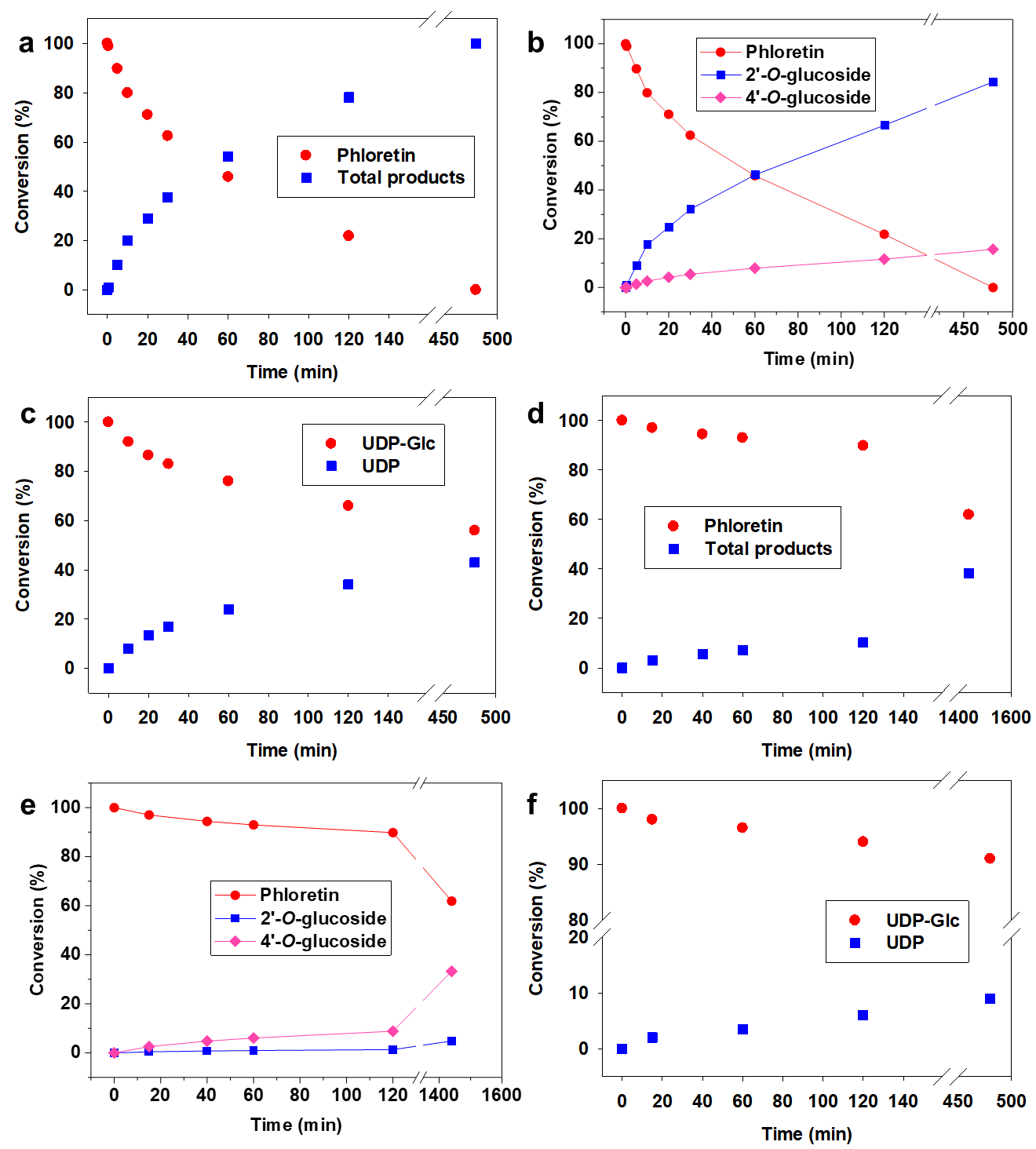


**Supplementary Figure 10**. Time courses of phloretin/total products, phloretin/individual products and UDP-Glc/UDP in UGT/phloretin reactions: UGT72D1 (**a-c**), and UGT84A119 (**d-f**). Reactions (100 μL) contained 1.0 mM phloretin, 2.0 mM UDP-Glc and 0.50 mg mL^-1^ enzymes in potassium phosphate buffer (50 mM, pH 8.0).

**
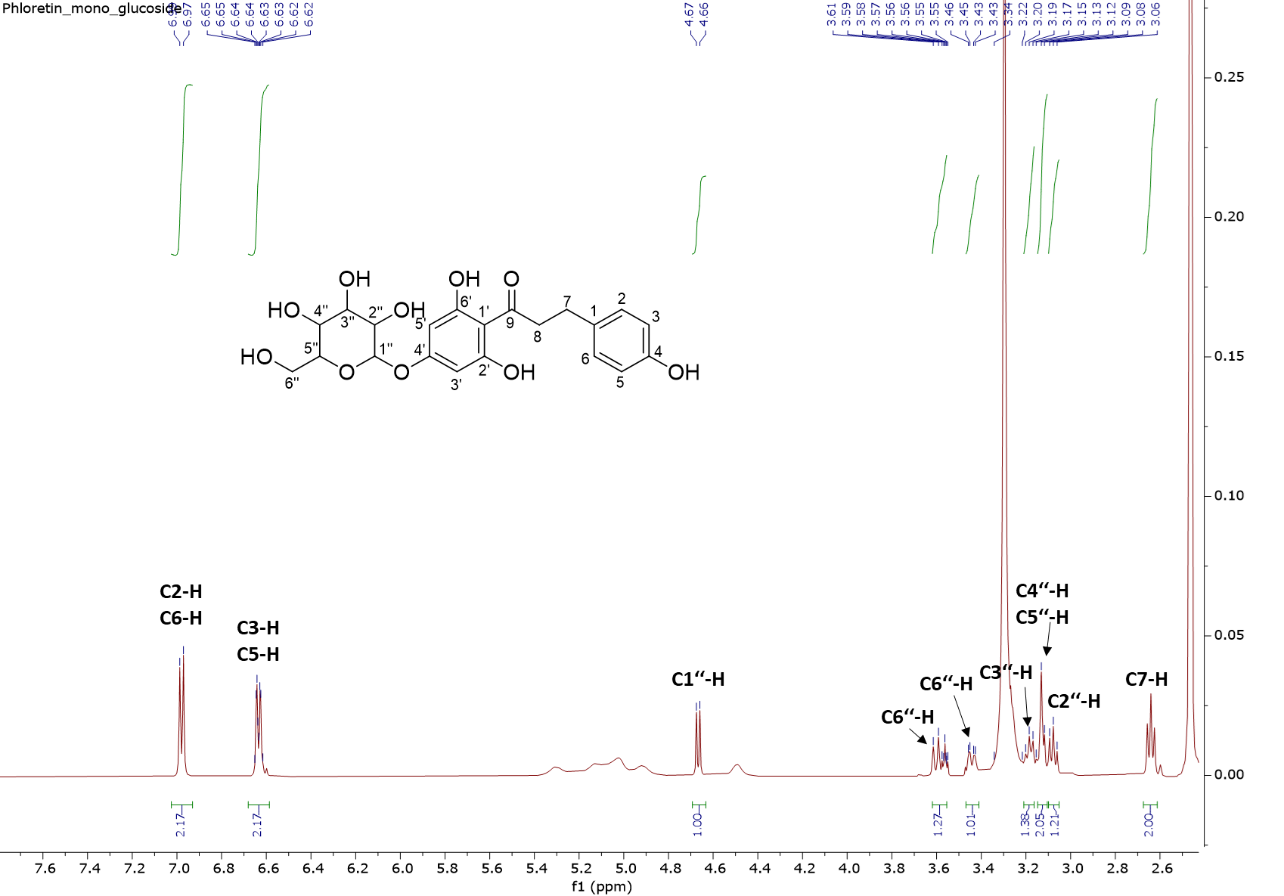
**

**Supplementary Figure 11**. ^1^H NMR spectrum (500 MHz, D_2_O) of isolated phloretin 4′-*O*-glucoside: δ 6.98 ppm (d, 2H), 6.63 ppm (d, 2H), 4.66 ppm (d, 1H), 3.58 ppm (m, 1H), 3.44 ppm (m, 1H), 3.19 ppm (m, 1H), 3.13 ppm (m, 2H), 3.08 ppm (t, 1H), 2.63ppm (t, 2H). The signal from C8-H is under the signal of water. Absence of the singlet from C3′+C5′ is attributed to an interference from a paramagnetic ion interacting with the phenolic hydroxyl groups of ring A.

**
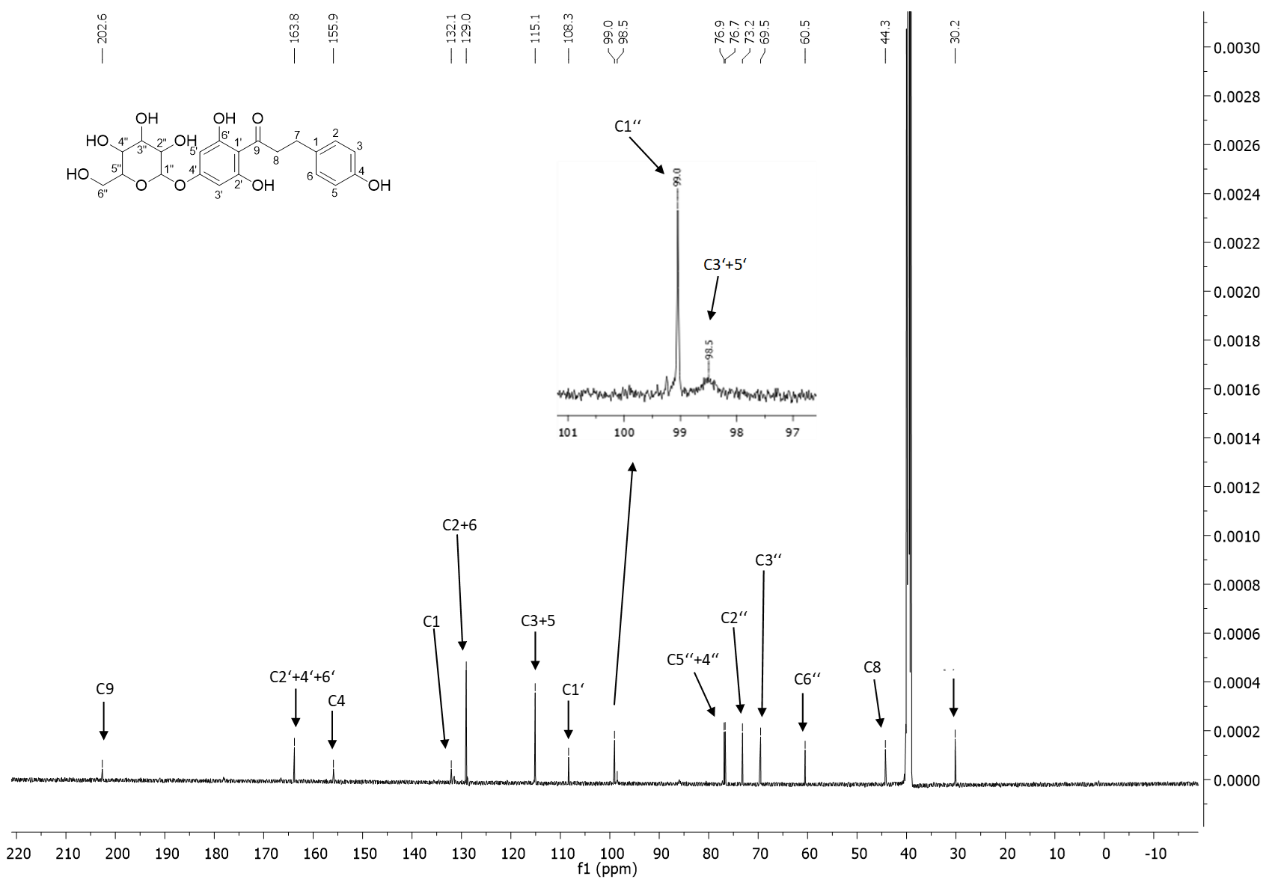
**

**Supplementary Figure 12**. ^13^C NMR spectrum (500 MHz, D_2_O) of phloretin 4′-*O*-glucoside. Broadening of the signal from C3′+C5′ is attributed to an interference from a paramagnetic ion interacting with the phenolic hydroxyl groups of ring A.

**
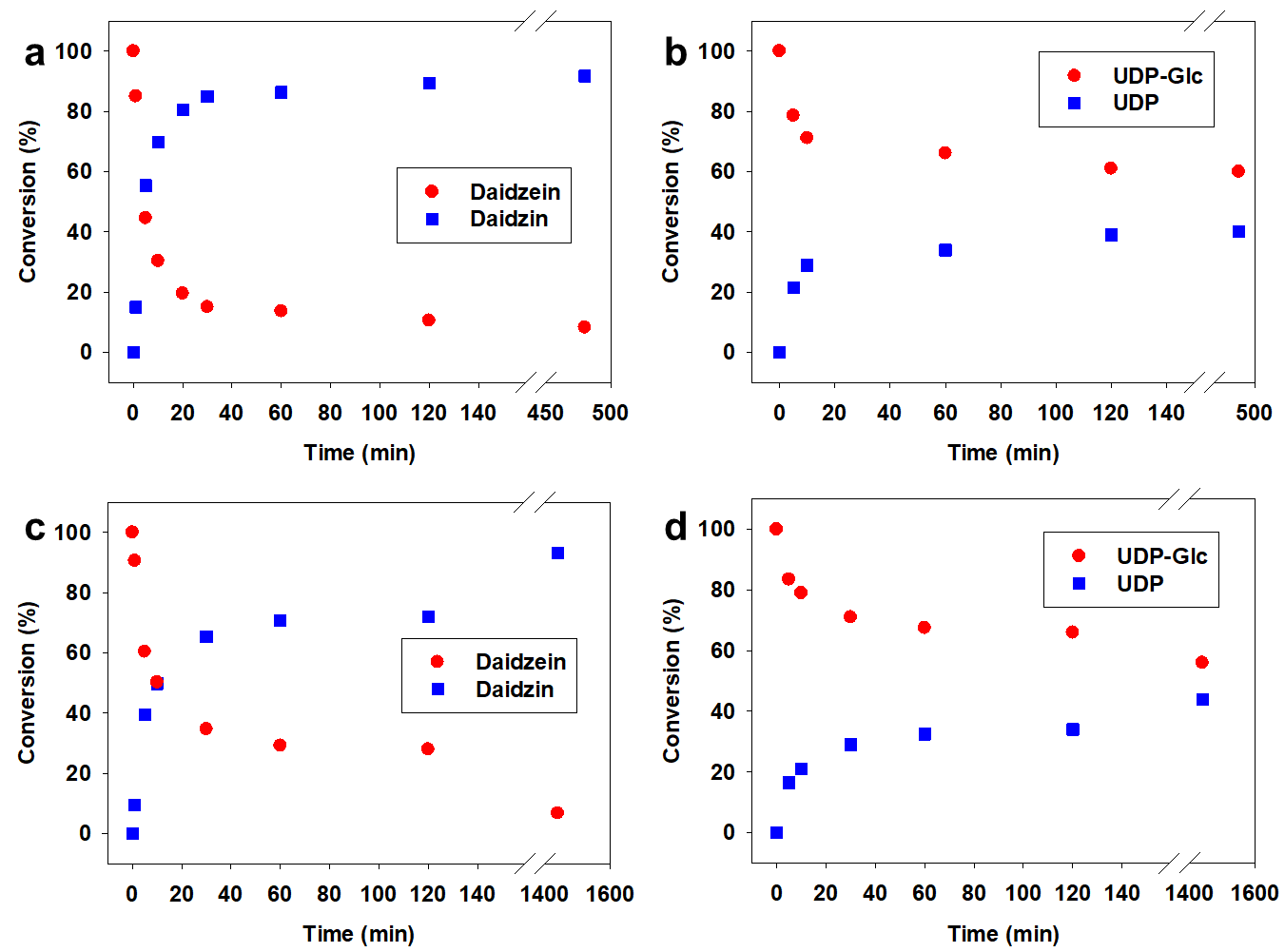
**

**Supplementary Figure 13**. Time courses of daidzein/products and UDP-Glc/UDP in UGT/daidzein reactions: UGT84A49 (**a**, **b**) and UGT84A119 (**c**, **d**). Reactions (100 μL) contained 1.0 mM daidzein, 2.0 mM UDP-Glc and 0.50 mg mL^-1^ enzymes in potassium phosphate buffer (50 mM, pH 8.0).


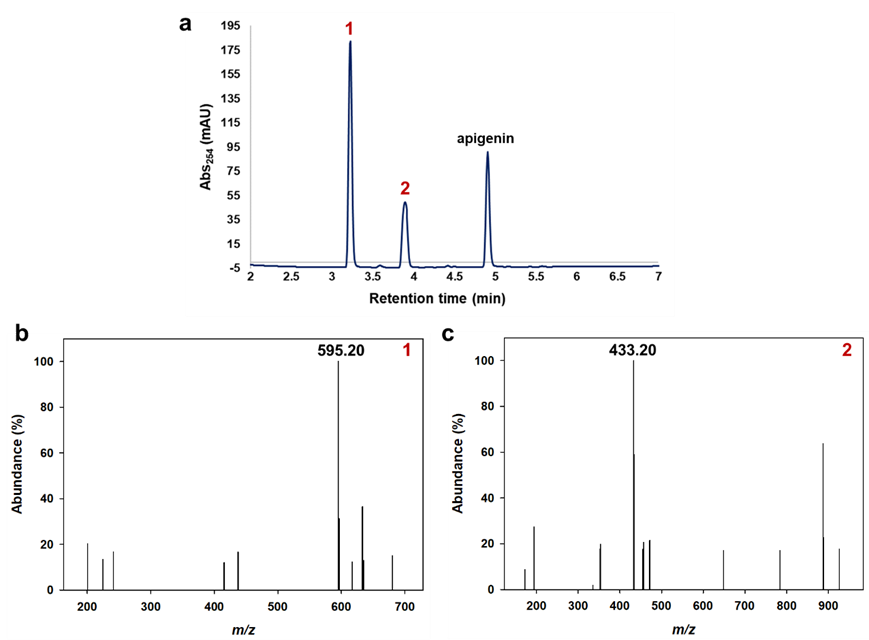


**Supplementary Figure 14**. Reverse phase HPLC-UV/MS analysis of the UGT84A49 reaction with apigenin at 8 h time point. **a**. HPLC chromatogram of apigenin 7,4′-di-*O*-glucoside (peak 1), apigenin 7-*O*-glucoside (peak 2), apigenin 4′-*O*-glucoside (peak 2) and apigenin. Note: Apigenin mono-*O*-glucosides co-elute in peak 2. **b**. Mass spectrum of peak 1 (apigenin 7,4′-di-*O*-glucoside) from chromatogram in **a**; calculated [M+H^+^]^+^ = 595.15, found: 595.20. **c**. Mass spectrum of peak 2 (apigenin 7-*O*-glucoside, apigenin 4′-*O*-glucoside) from chromatogram in **a**, calculated [M+H^+^]^+^ = 433.11, found: 433.20.


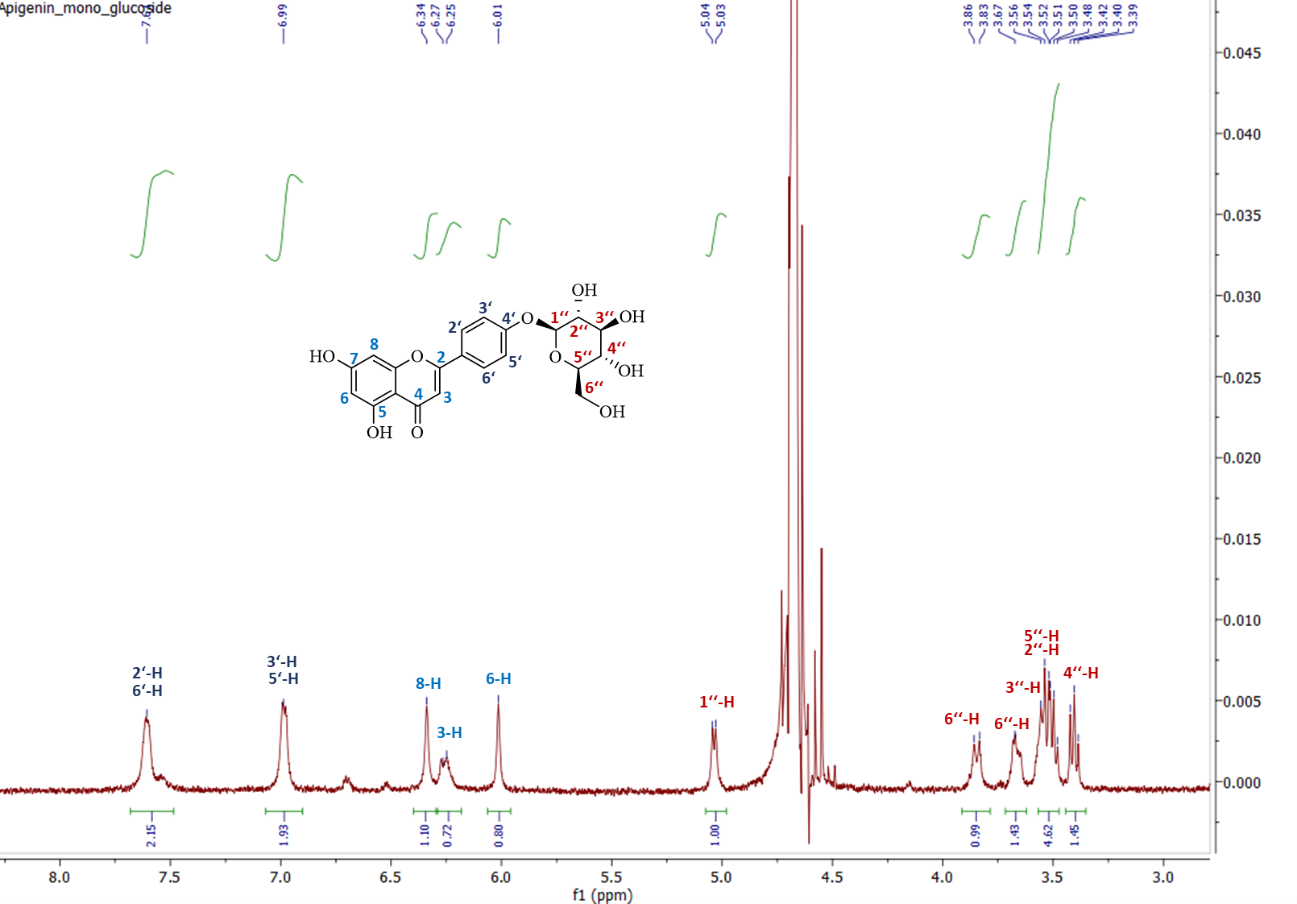


**Supplementary Figure 15**. ^1^H NMR spectrum (500 MHz, 20% DMSO-d6, D_2_O) of isolated apigenin 4′-*O*-glucoside: δ 7.62 ppm (d, 2H), 6.99 ppm (d, 2H), 6.34 ppm (s, 1H), 6.26 ppm (s, 1H), 6.01 ppm (s, 1H), 5.03 ppm (d, 1H), 3.85 ppm (d, 1H), 3.67 ppm (m, 1H) 3.52 ppm (m, 3H), 3.40 ppm (t, 1H).


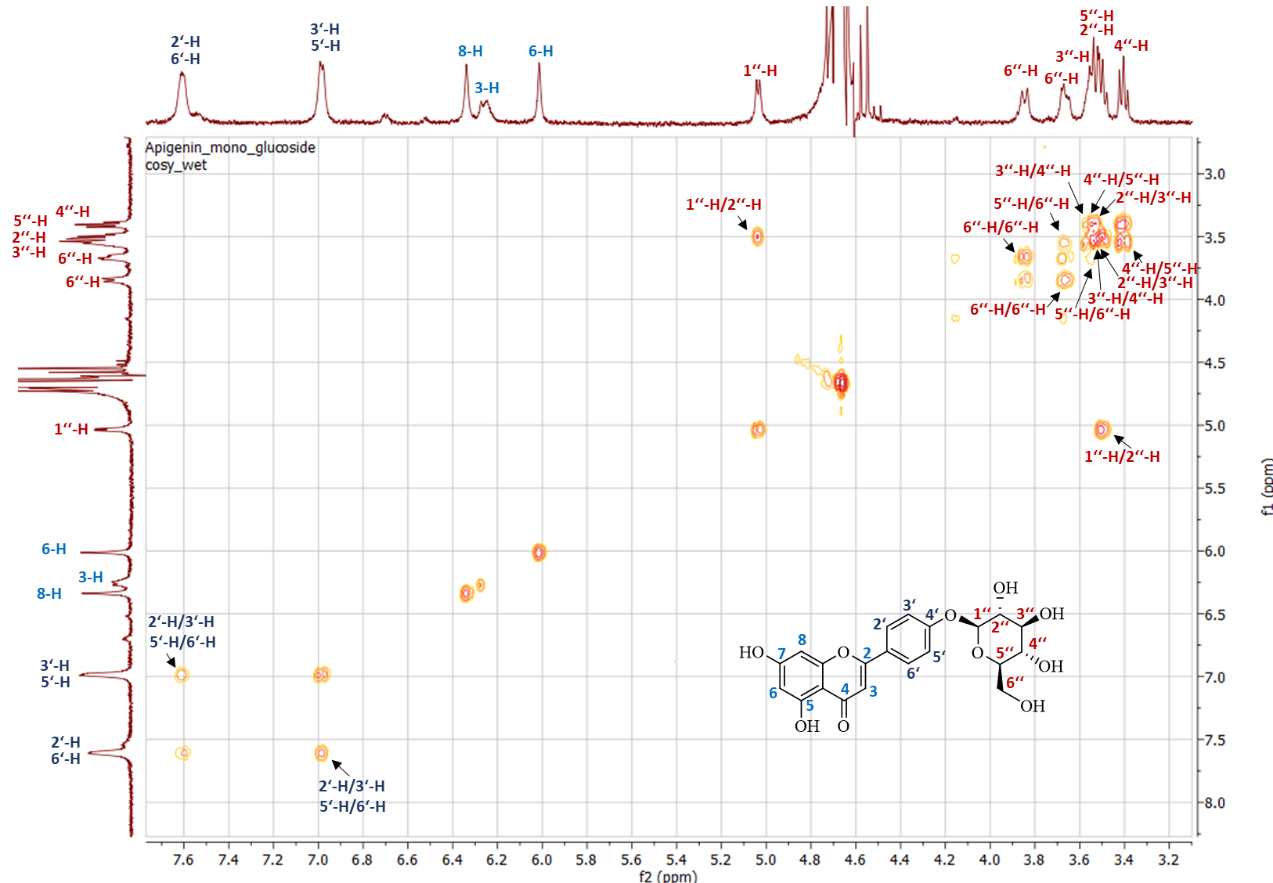


**Supplementary Figure 16**. Correlated spectroscopy (COSY) analysis of apigenin 4′-*O*-glucoside (500 MHz, 20% DMSO-d6, D_2_O).

**
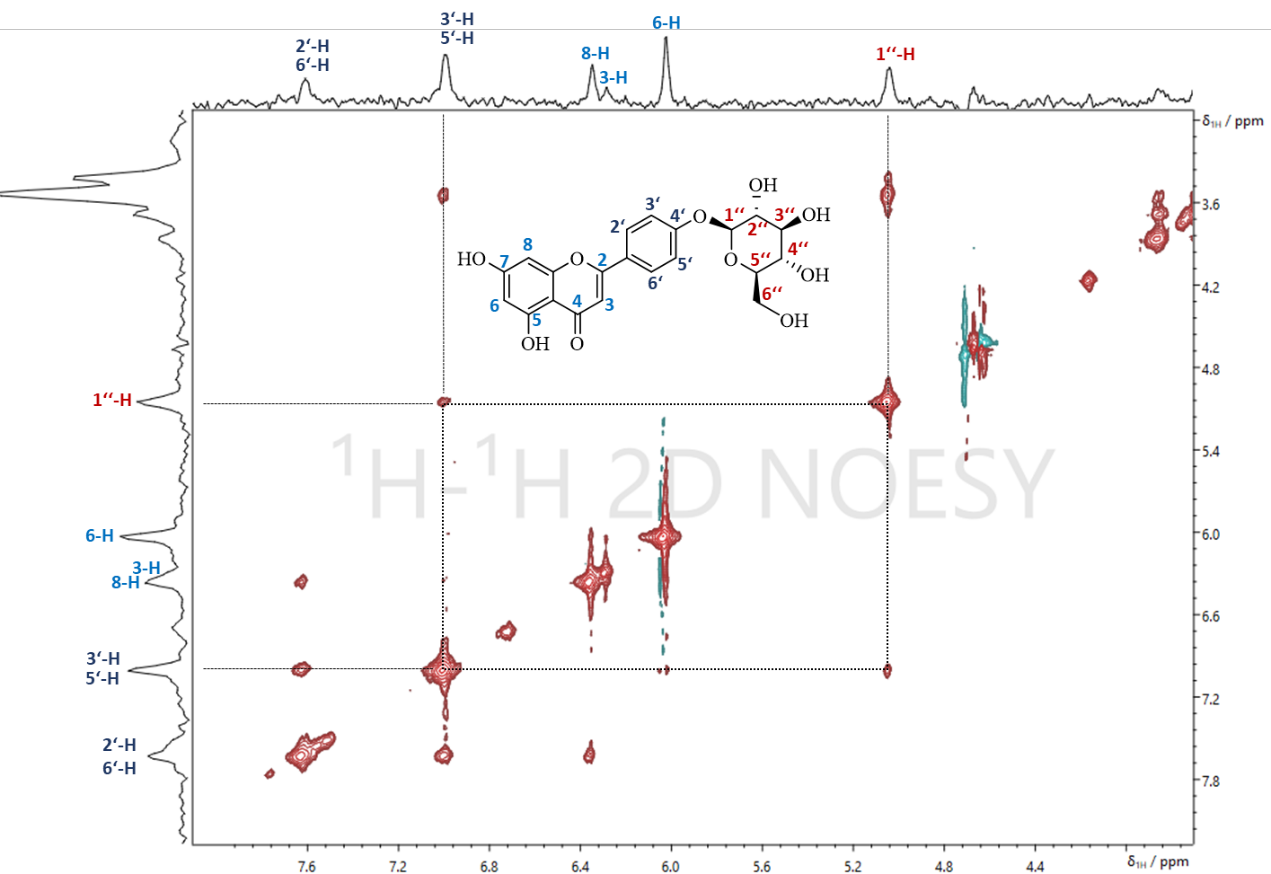
**

**Supplementary Figure 17**. 2D NOE spectroscopy (2D NOESY) analysis of apigenin 4′-*O*-glucoside (500 MHz, 20% DMSO-d6, D_2_O). A crosspeak is seen between 1′′-H (anomeric proton) and 3′-H/5′-H (aromatic protons from ring B in ortho position to glucose).


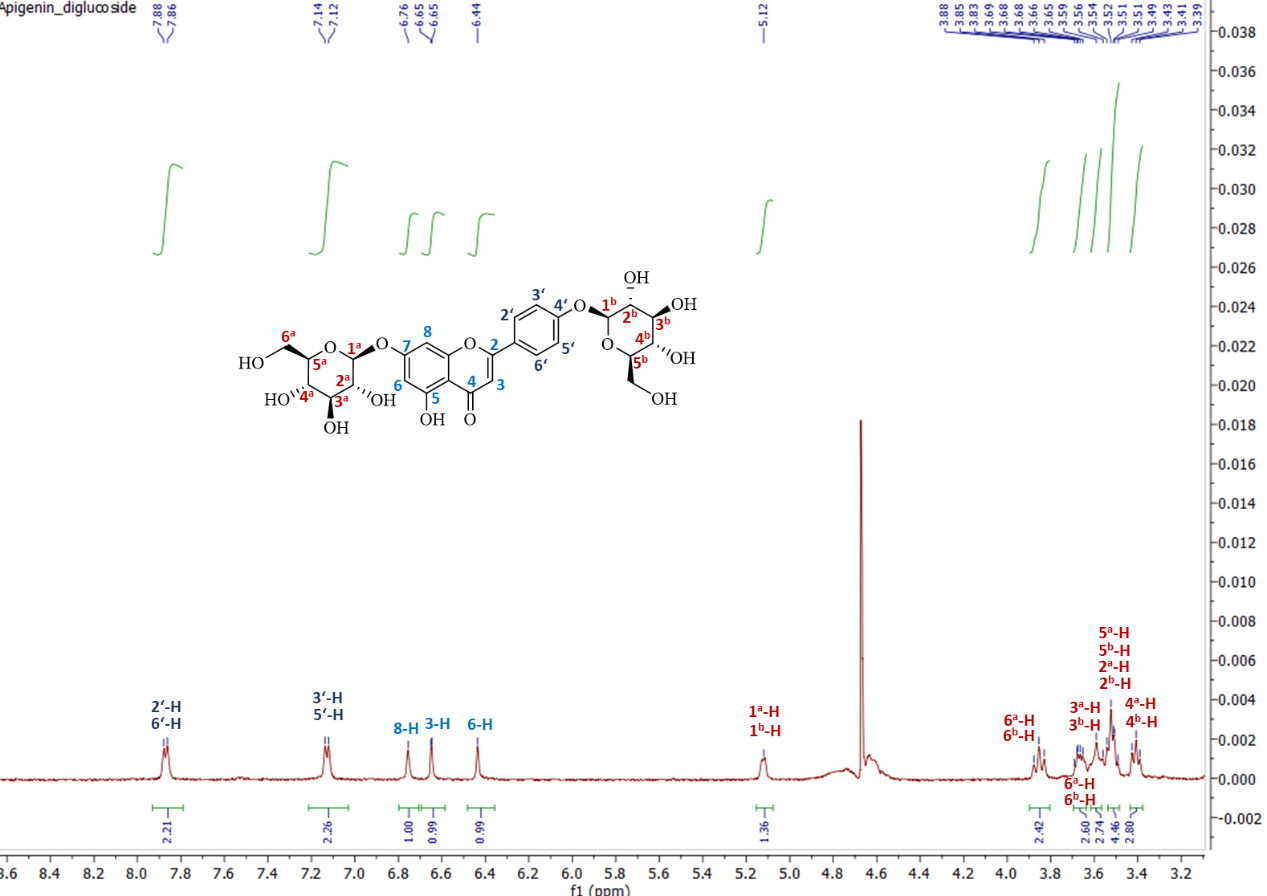


**Supplementary Figure 18**. ^1^H NMR spectrum (500 MHz, 15% DMSO-d6, D_2_O) of isolated apigenin 7,4′-di-*O*-glucoside: δ 7.87 ppm (d, 2H), 7.13 ppm (d, 2H), 6.76 ppm (s, 1H), 6.65 ppm (s, 1H), 6.44 ppm (s, 1H), 5.12 ppm (d, 2H), 3.85 ppm (t, 2H), 3.68 ppm (m, 2H) 3.59 ppm (m, 2H), 3.51 ppm (m, 4H), 3.40 ppm (t, 2H).


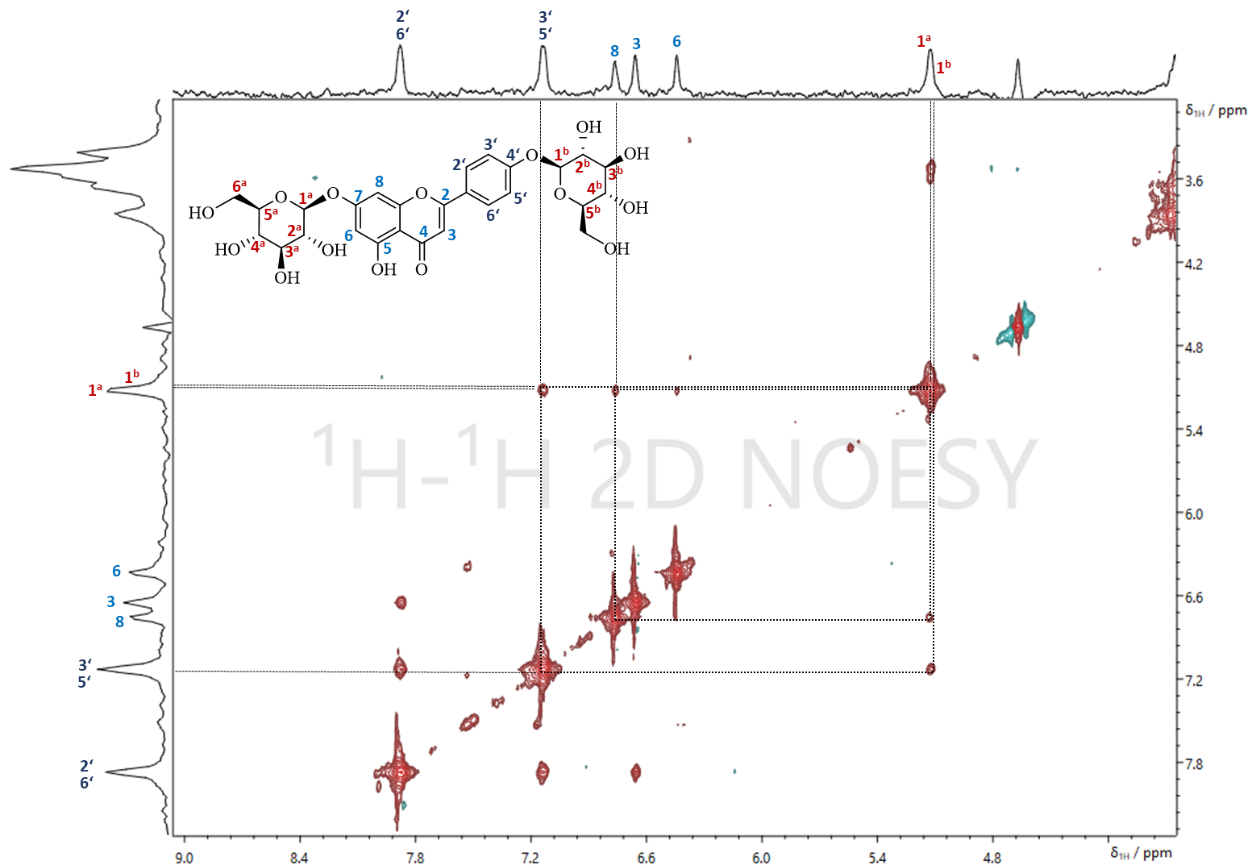


**Supplementary Figure 19**. 2D NOESY analysis of apigenin 7,4′-di-*O*-glucoside (500 MHz, 15% DMSO-d6, D_2_O). Crosspeaks are seen between 1^a^-H (anomeric proton from glucose at 7-*O*) and 8-H, and between 1^b^-H (anomeric proton from glucose at 4′-*O*) and 3′-H/5′-H.

**
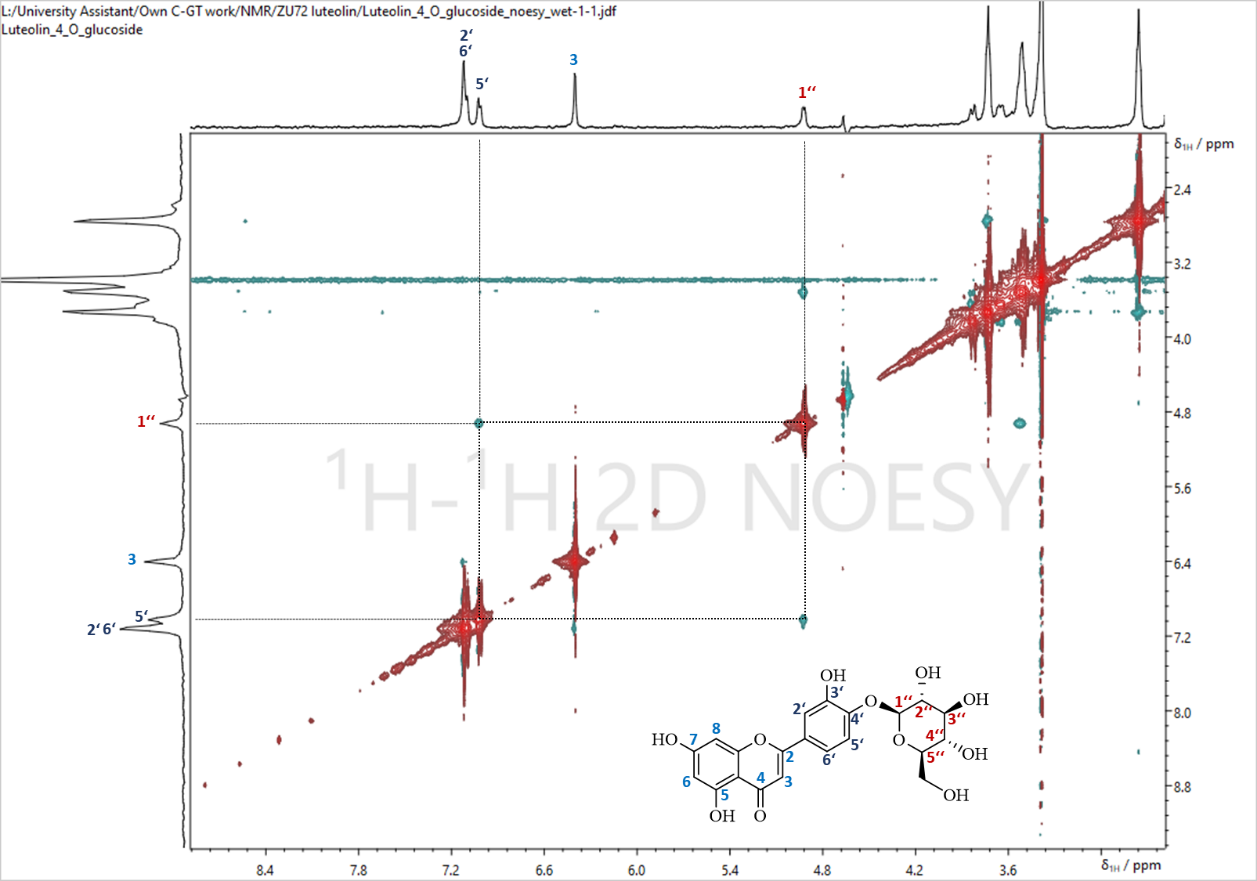
**

**Supplementary Figure 20**. 2D NOESY analysis of luteolin 4′-*O*-glucoside (500 MHz, 15% DMSO-d6, D_2_O). A crosspeak is seen between 1′′-H (anomeric proton) and 5′-H (ring B aromatic proton in ortho position to glucose). The missing signals from 6-H and 8-H are attributed to an interference from a paramagnetic ion interacting with the hydroxyl groups of ring A.

**
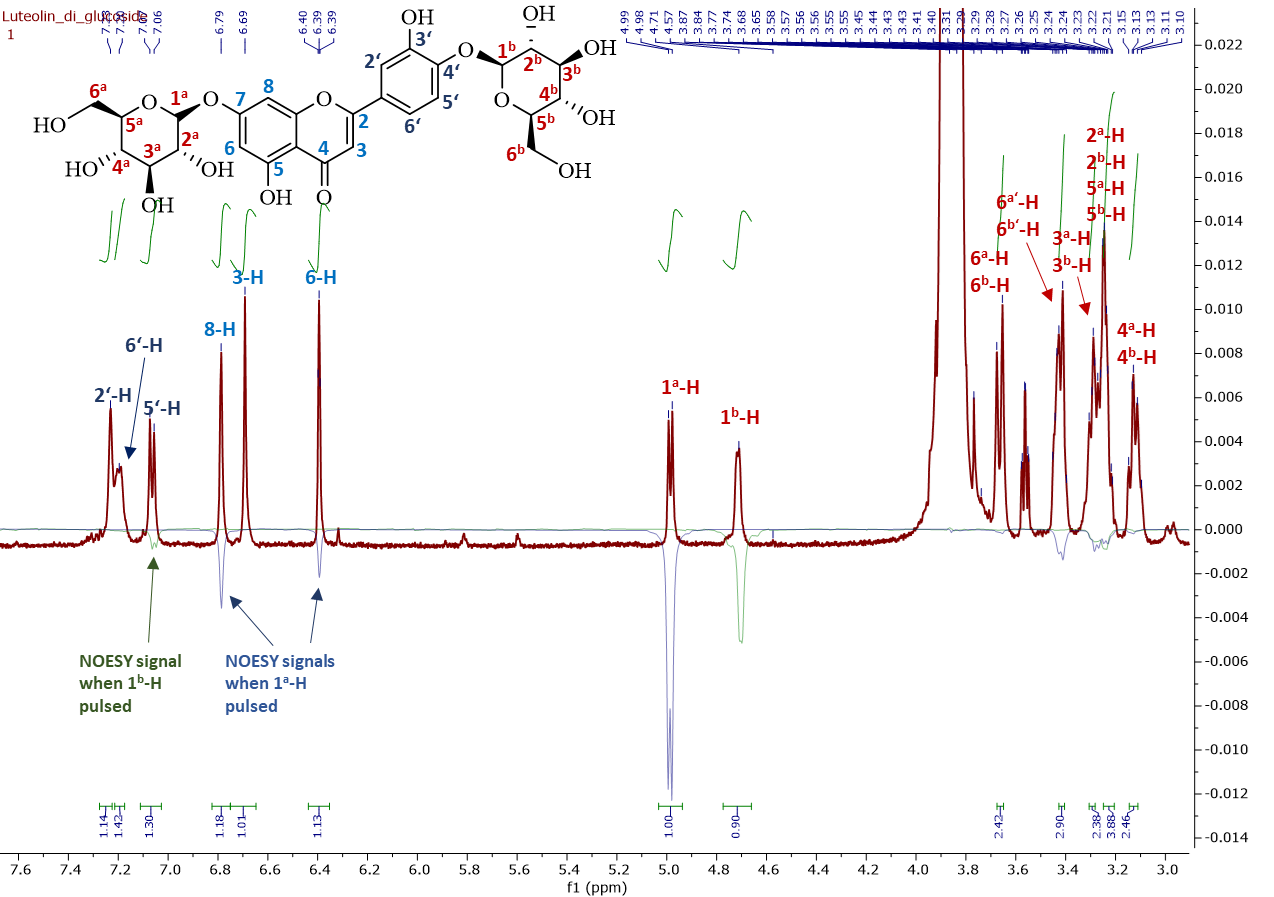
**

**Supplementary Figure 21**. Overlay of 1D NOESY and ^1^H NMR spectra (500 MHz, 10% DMSO-d6, D_2_O) of isolated luteolin-7,4′-di-*O*-glucoside. δ 7.25 ppm (s, 1H), 7.20 ppm (d, 1H), 7.06 ppm (d, 1H), 6.79 ppm (s, 1H), 6.69 ppm (s, 1H), 6.39 ppm (s, 1H), 4.98 ppm (d, 1H), 4.71 ppm (s, 1H), 3.65 ppm (d, 2H), 3.45 ppm (m, 2H), 3.25 ppm (m, 6H), 3.13 ppm (t, 1H). 1D NOESY spectrum shows the appearing signals 8-H and 6-H upon pulsing the anomeric proton 1^a^-H, and the signal 5′-H upon pulsing the anomeric proton 1^b^-H.

**
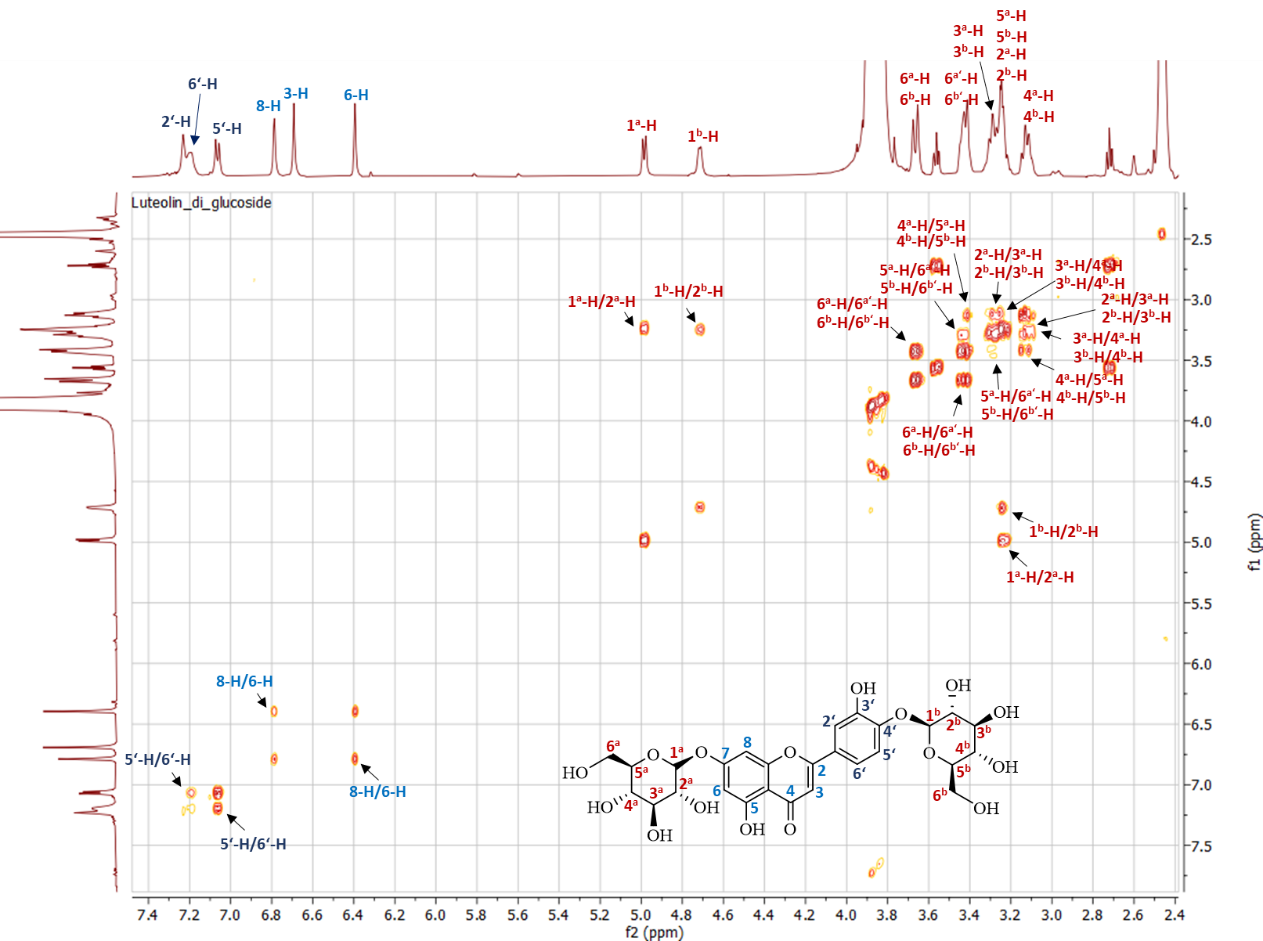
**

**Supplementary Figure 22**. COSY analysis of luteolin-7,4′-di-*O*-glucoside (500 MHz, 10% DMSO-d6, D_2_O).


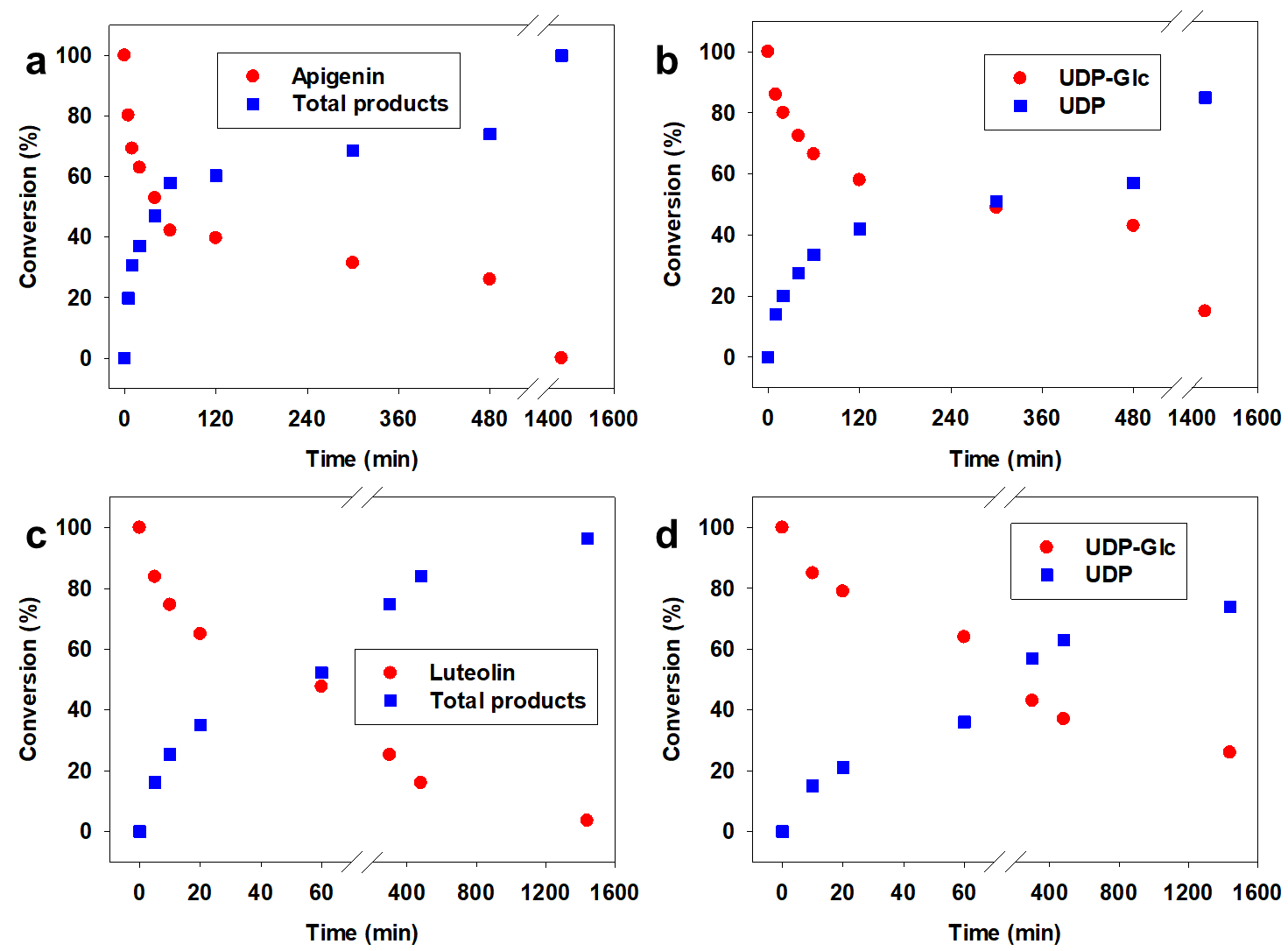


**Supplementary Figure 23**. Time courses of acceptor substrates/total products and UDP-Glc/UDP in UGT84A49 reactions toward apigenin (**a**, **b**) and luteolin (**c**, **d**). Reactions (100 μL) contained 1.0 mM apigenin or luteolin, 2.0 mM UDP-Glc and 0.50 mg mL^-1^ UGT84A49 in potassium phosphate buffer (50 mM, pH 8.0).


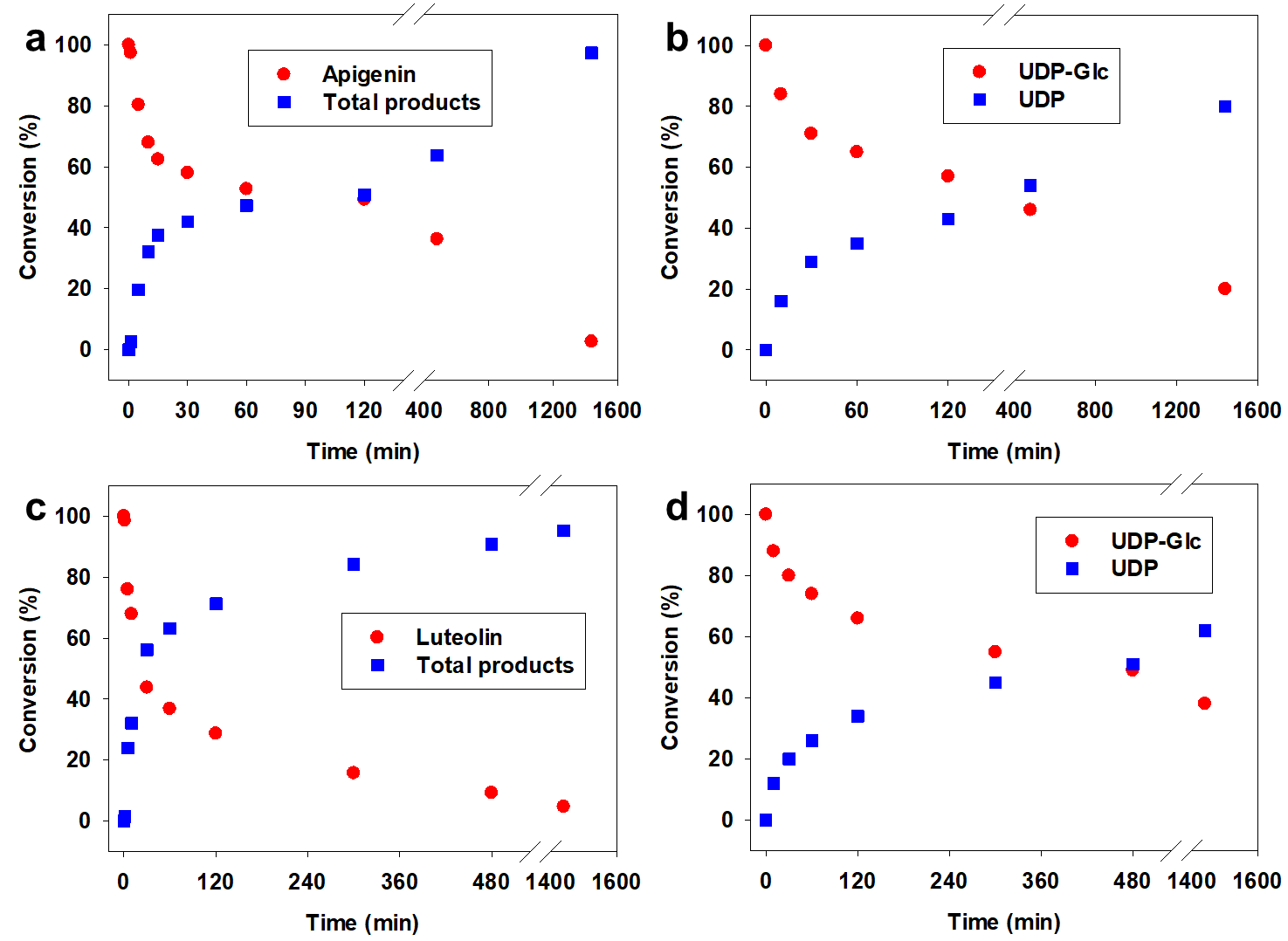


**Supplementary Figure 24**. Time courses of acceptor substrates/total products and UDP-Glc/UDP in UGT84A119 reactions toward apigenin (**a**, **b**) and luteolin (**c**, **d**). Reactions (100 μL) contained 1.0 mM apigenin or luteolin, 2.0 mM UDP-Glc and 0.50 mg mL^-1^ UGT84A119 in potassium phosphate buffer (50 mM, pH 8.0).

**
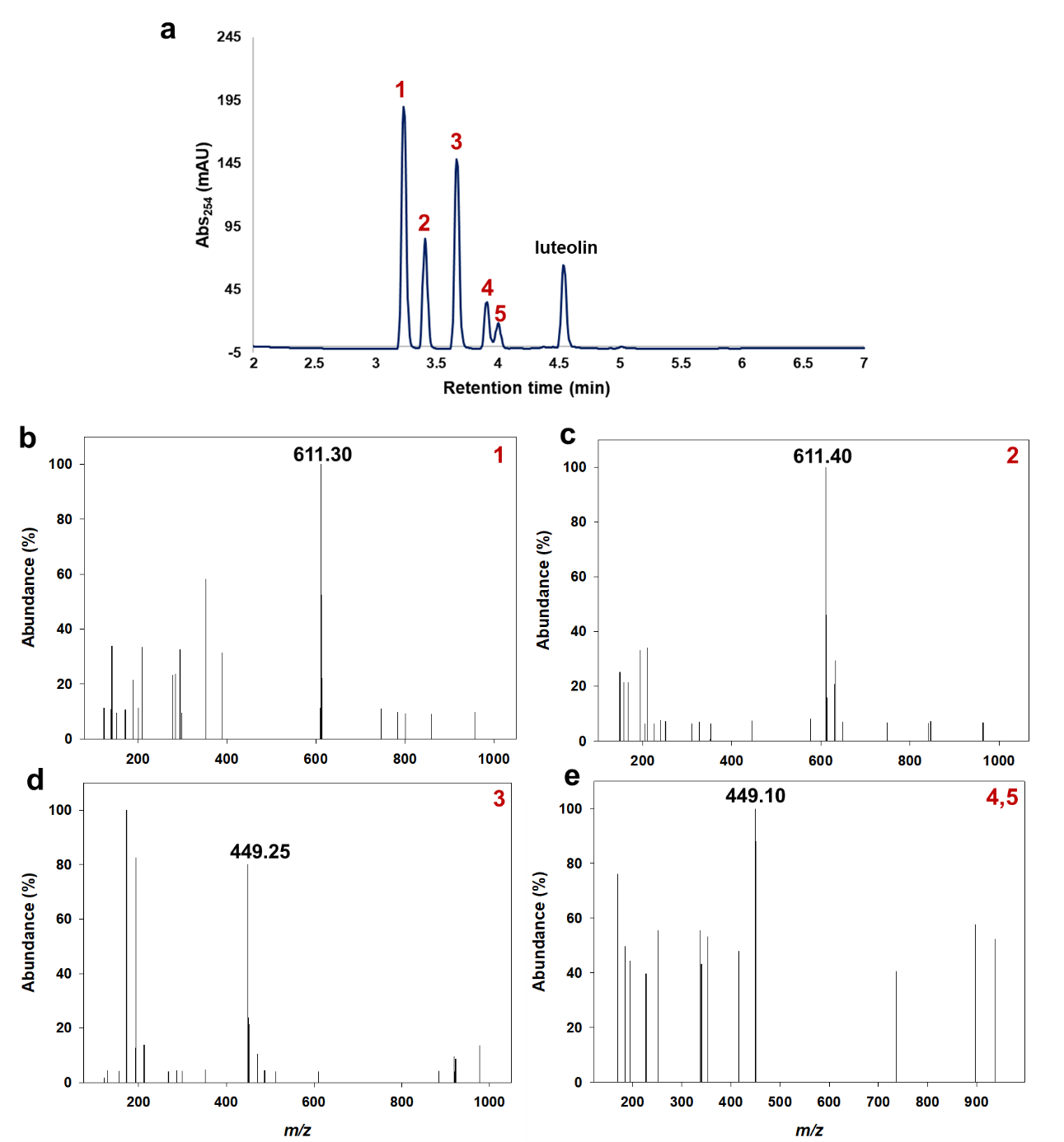
**

**Supplementary Figure 25**. Reverse phase HPLC-UV/MS analysis of the UGT84A119 reaction with luteolin at 8 h time point. **a**. HPLC chromatogram of luteolin 7,4′-di-*O*-glucoside (peak 1), luteolin 7,3′-di-*O*-glucoside (peak 2), luteolin 7-*O*-glucoside (peak 3), luteolin 4′-*O*-glucoside (peak 4), luteolin 3′-*O*-glucoside (peak 5) and luteolin. **b**. Mass spectrum of peak 1 (luteolin 7,4′-di-*O*-glucoside) from chromatogram in **a**; calculated [M+H^+^]^+^ = 611.15, found: 611.30. **c**. Mass spectrum of peak 2 (luteolin 7,3′-di-*O*-glucoside) from chromatogram in **a**, calculated [M+H^+^]^+^ = 611.15, found: 611.40. **d**. Mass spectrum of peak 3 (luteolin 7-*O*-glucoside) from chromatogram in **a**, calculated [M+H^+^]^+^ = 449.10, found: 449.25. **d**. Mass spectrum of peaks 4 and 5 (luteolin 4′-*O*-glucoside, luteolin 3′-*O*-glucoside) from chromatogram in **a**, calculated [M+H^+^]^+^ = 449.10, found: 449.10.

**
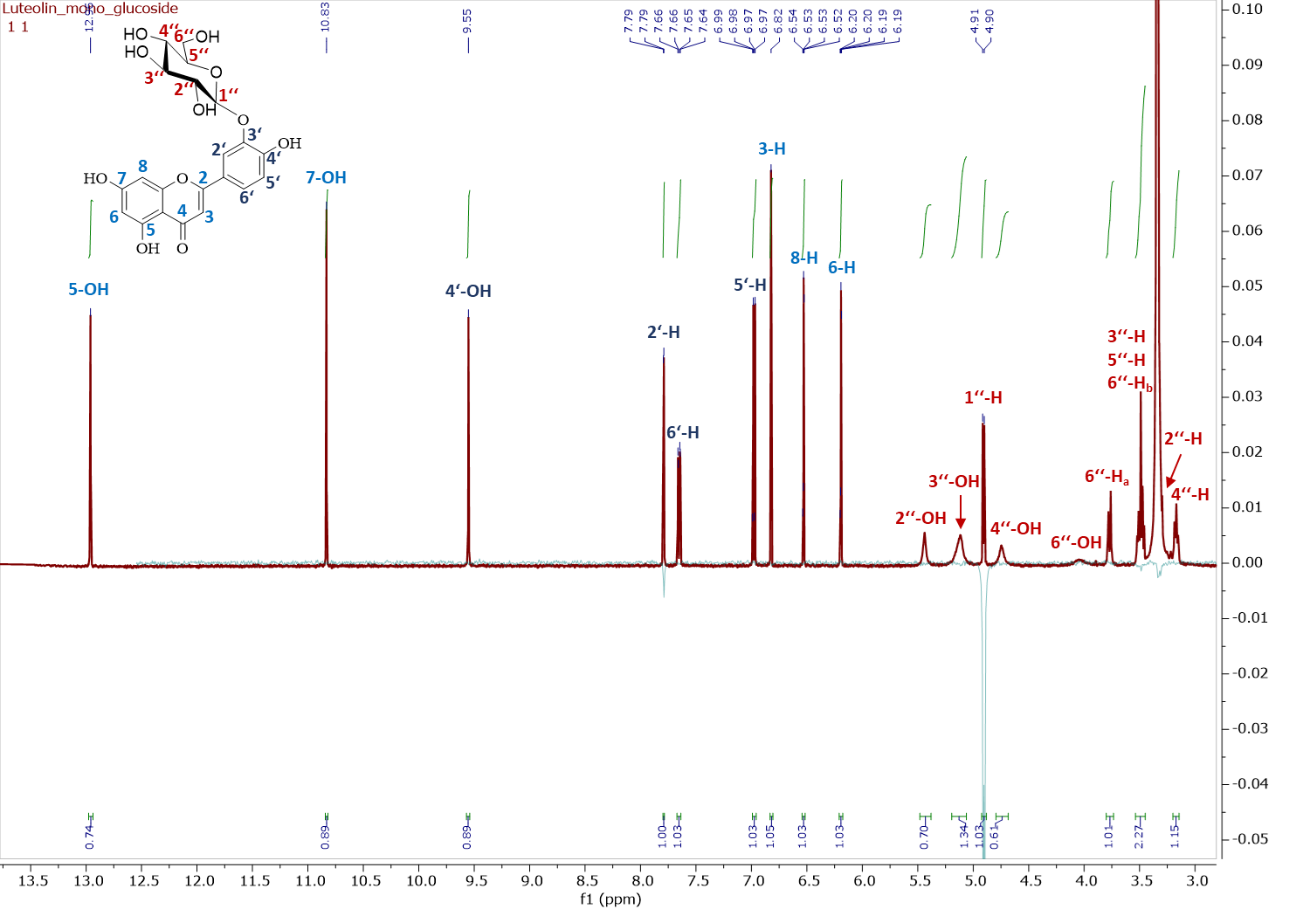
**

**Supplementary Figure 26**. Overlay of 1D NOESY and ^1^H NMR spectra (500 MHz, DMSO-d6) of isolated luteolin 3′-*O*-glucoside. δ 12.96 ppm (-OH), 10.83 ppm (-OH), 9.55 ppm (-OH), 7.79 ppm (s, 1H), 7.65 ppm (d, 1H), 6.82 ppm (s, 1H), 6.53 ppm (s, 1H), 6.19 ppm (s, 1H), 5.45 ppm (s, OH), 5.15 ppm (s, -OH), 4.90 ppm (d, 1H), 4.73 ppm (s, -OH), 4.10 ppm (s, -OH), 3.75 ppm (d, 1H), 3.50 ppm (m, 3H), 3.30 ppm (m, 1H), 3.20 ppm (t, 1H). 1D NOESY spectrum shows the appearing signal of 2′-H upon pulsing the anomeric proton 1′′-H.


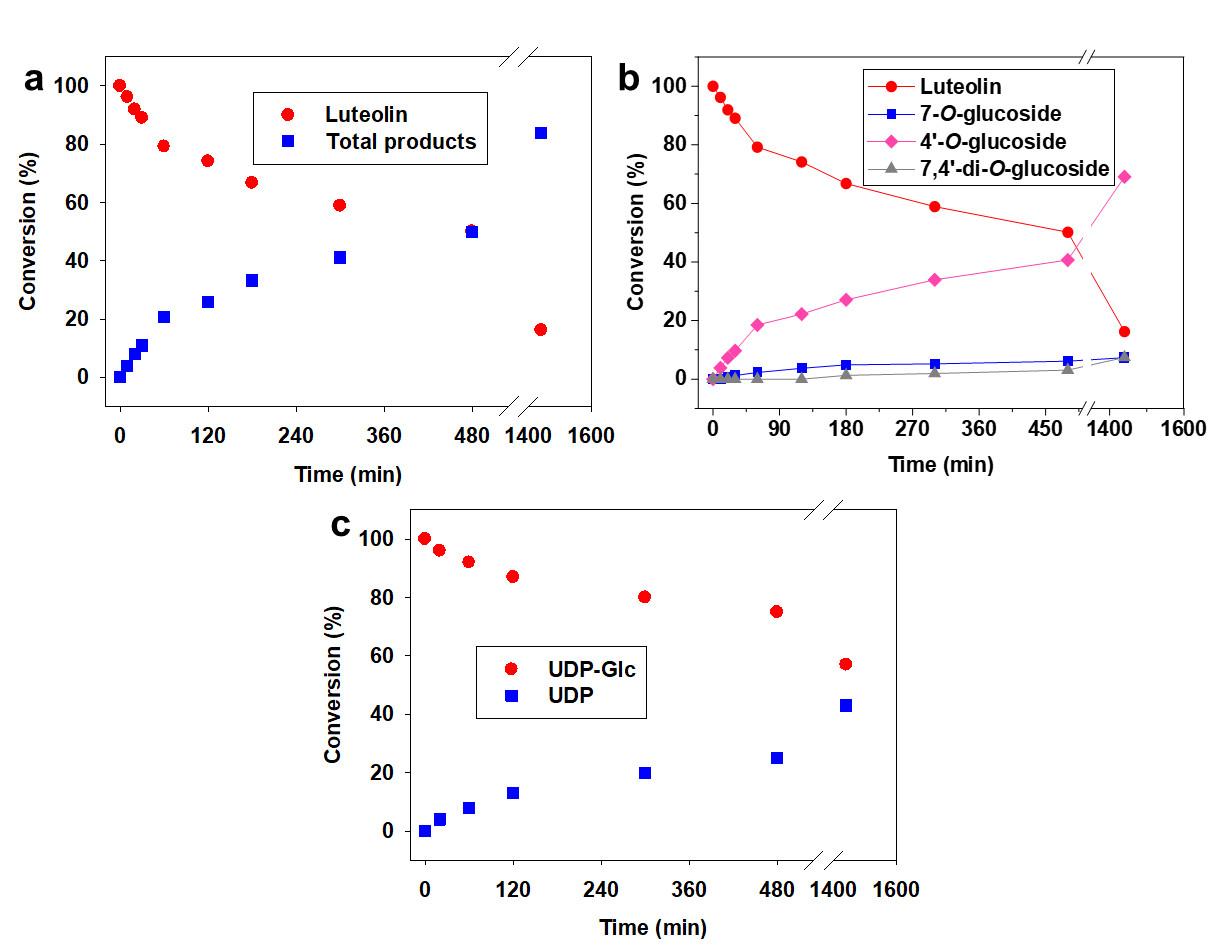


**Supplementary Figure 27**. Time courses of luteolin/total products (**a**), luteolin/individual products (**b**) and UDP-Glc/UDP (**c**) in UGT72D7 reactions toward luteolin. Reactions (100 μL) contained 1.0 mM luteolin, 2.0 mM UDP-Glc and 0.50 mg mL^-1^ UGT72D7 in potassium phosphate buffer (50 mM, pH 8.0).


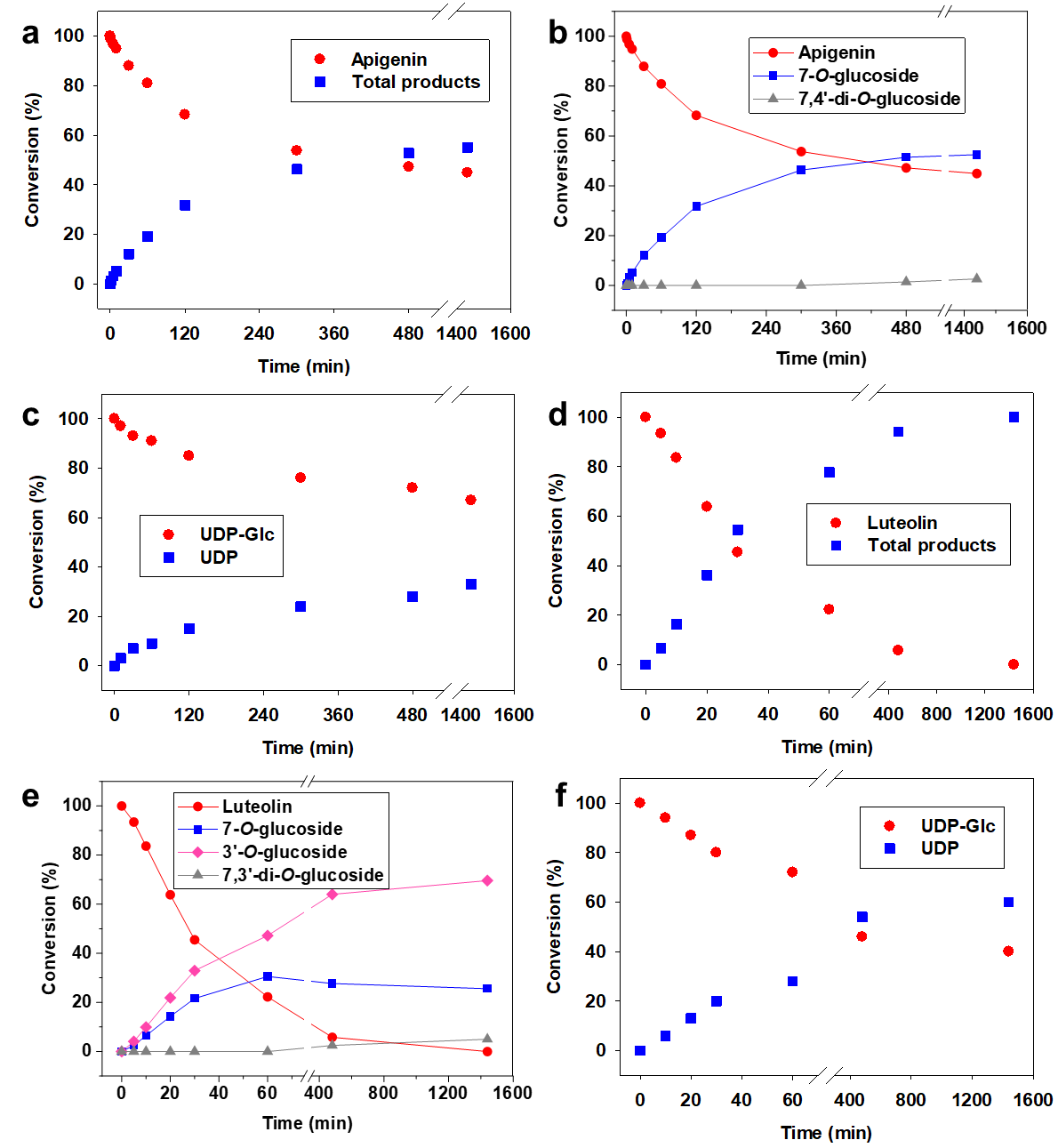


**Supplementary Figure 28**. Time courses of acceptor substrates/total products, acceptor substrates/individual products and UDP-Glc/UDP in UGT72D1 reactions. **a**. Apigenin/total products, **b**. apigenin/individual products, and **c**. UDP-Glc/UDP in UGT72D1/apigenin reactions. **d**. Luteolin/total products, **e**. luteolin/individual products, and **f**. UDP-Glc/UDP in UGT72D1/luteolin reactions. Reactions (100 μL) contained 1.0 mM apigenin or luteolin, 2.0 mM UDP-Glc and 0.50 mg mL^-1^ UGT72D1 in potassium phosphate buffer (50 mM, pH 8.0).

**Supplementary Figure 29**. Proposed reaction mechanism for an inverting *O*-glycosyltransferase (OGT) from the UGT family^2^. B = active site base, typically histidine.


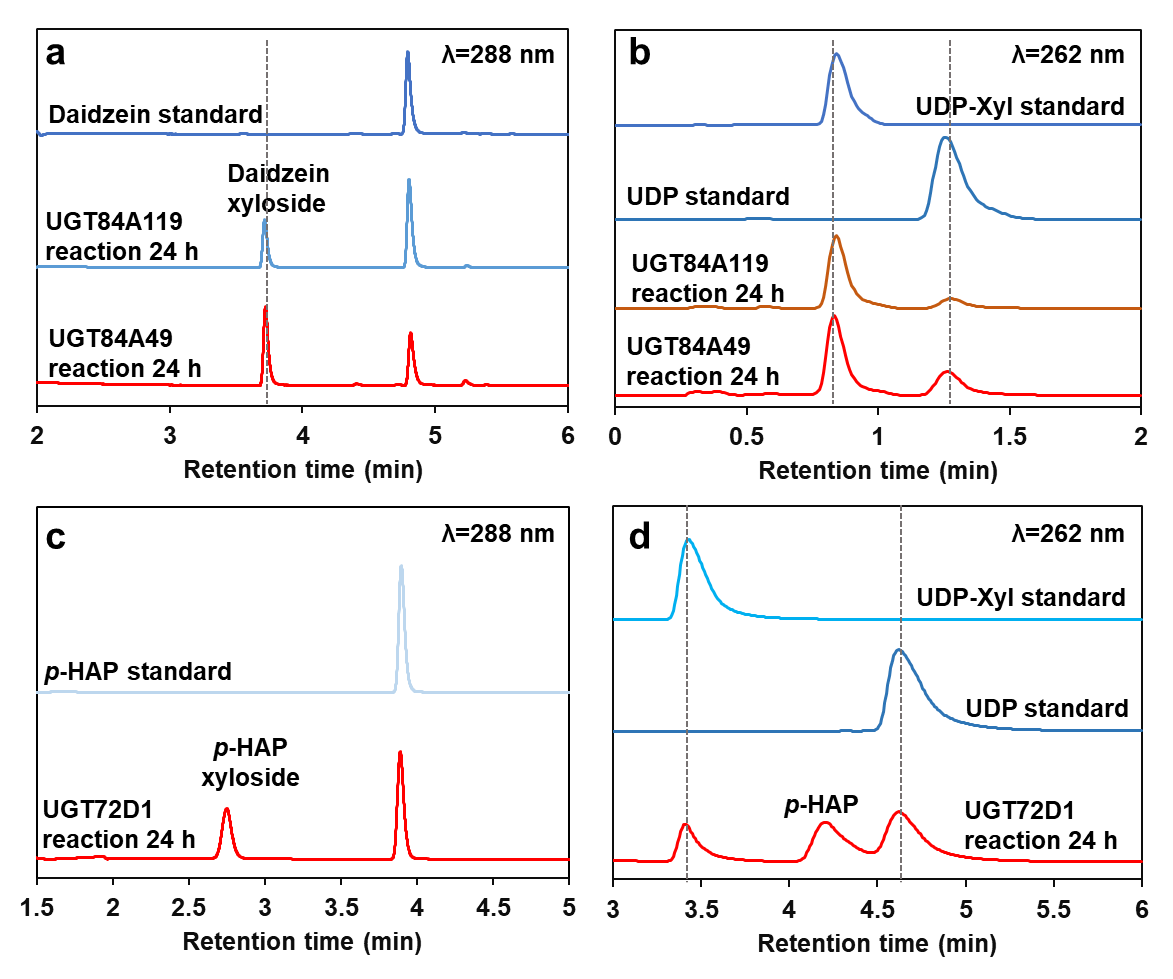


**Supplementary Figure 30**. Overlay of HPLC chromatograms for acceptor substrates/xylosides and UDP-Xyl/UDP in UGT reactions: UGT84A119 and UGT84A49 with daidzein/UDP-Xyl (**a**, **b**) and UGT72D1 with *p*-HAP/UDP-Xyl (**c**, **d**). Reactions (100 μL) contained 1.0 mM acceptor substrates, 2.0 mM UDP-Xyl and 0.50 mg mL^-1^ enzymes in potassium phosphate buffer (50 mM, pH 8.0).


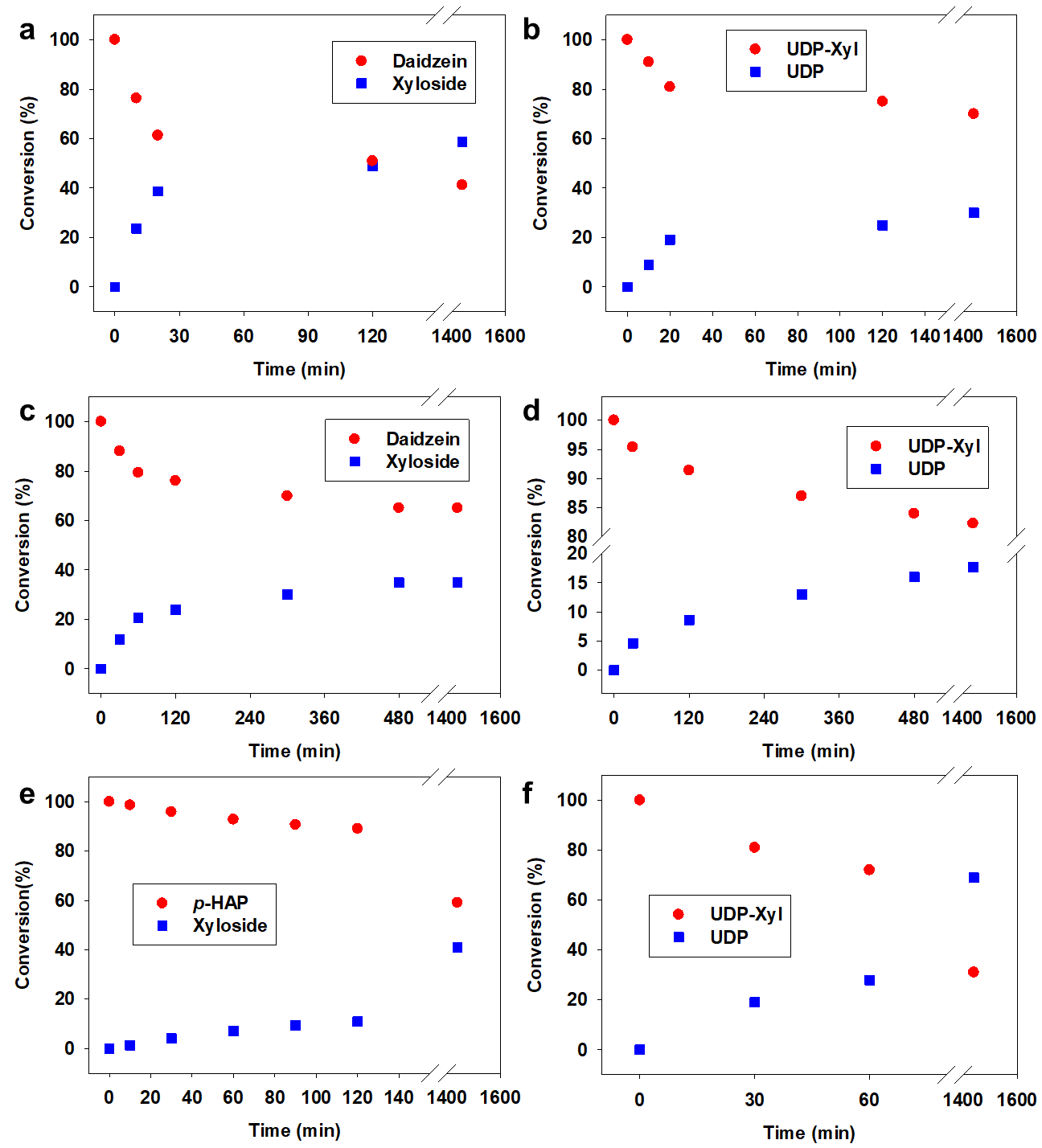


**Supplementary Figure 31**. Time courses of acceptor substrates/xylosides and UDP-Xyl/UDP in UGT reactions. **a**. Daidzein/xyloside, and **b**. UDP-Xyl/UDP in UGT84A49/daidzein/UDP-Xyl reactions. **c**. Daidzein/xyloside, and **d**. UDP-Xyl/UDP in UGT84A119/daidzein/UDP-Xyl reactions. **e**. *p*-HAP/xyloside, and **f**. UDP-Xyl/UDP in UGT72D1/*p*-HAP/UDP-Xyl reactions. Reactions (100 μL) contained 1.0 mM acceptor substrates, 2.0 mM UDP-Xyl and 0.50 mg mL^-1^ UGT in potassium phosphate buffer (50 mM, pH 8.0).


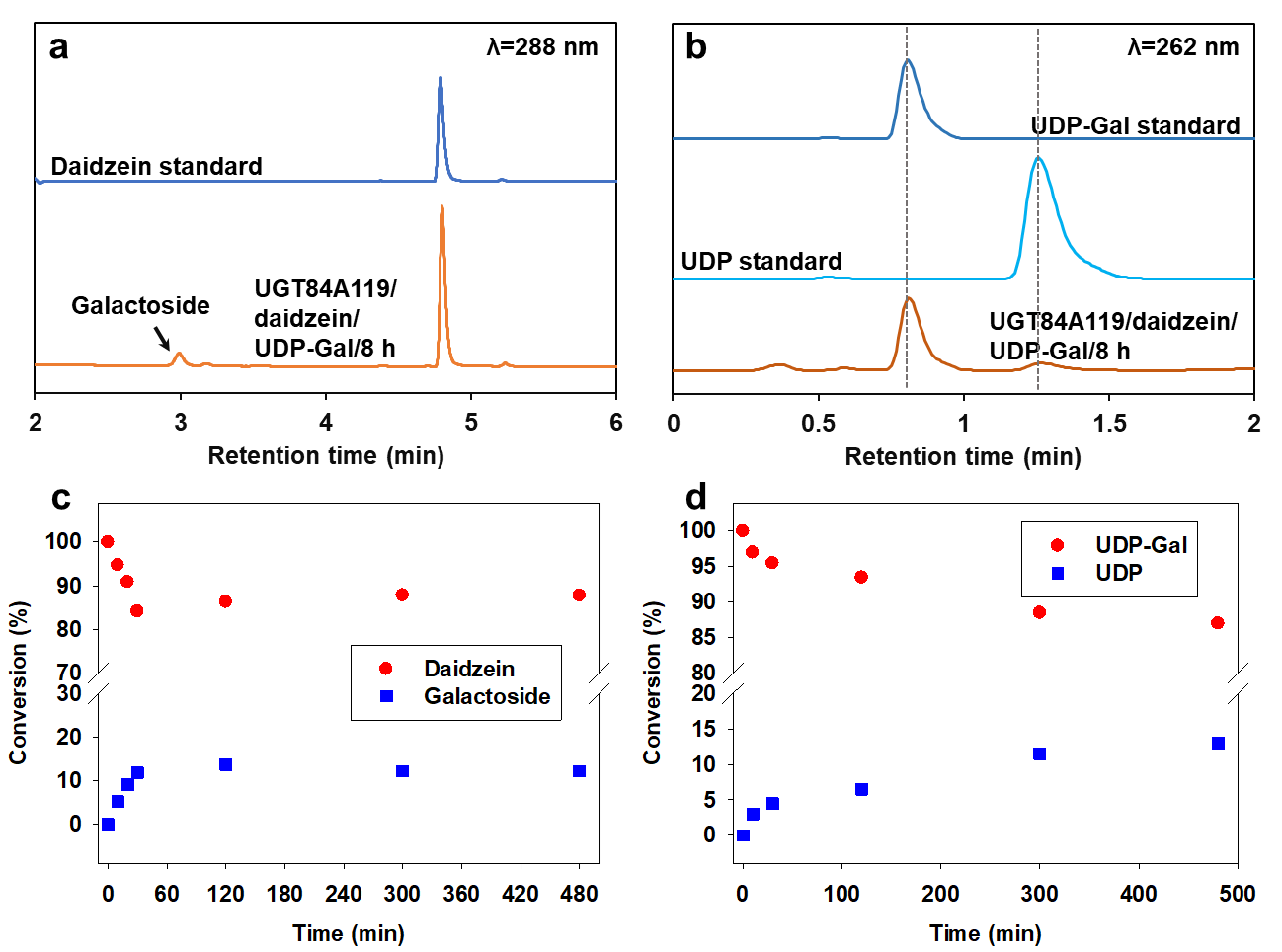


**Supplementary Figure 32**. Overlay of HPLC chromatograms for acceptor substrate/galactoside (**a**), UDP-Gal/UDP (**b**), and time courses of daidzein/galactoside (**c**) and UDP-Gal/UDP (**d**) in UGT84A119 reactions toward daidzein/UDP-Gal. Reactions (100 μL) contained 1.0 mM daidzein, 2.0 mM UDP-Gal and 0.50 mg mL^-1^ UGT84A119 in potassium phosphate buffer (50 mM, pH 8.0).


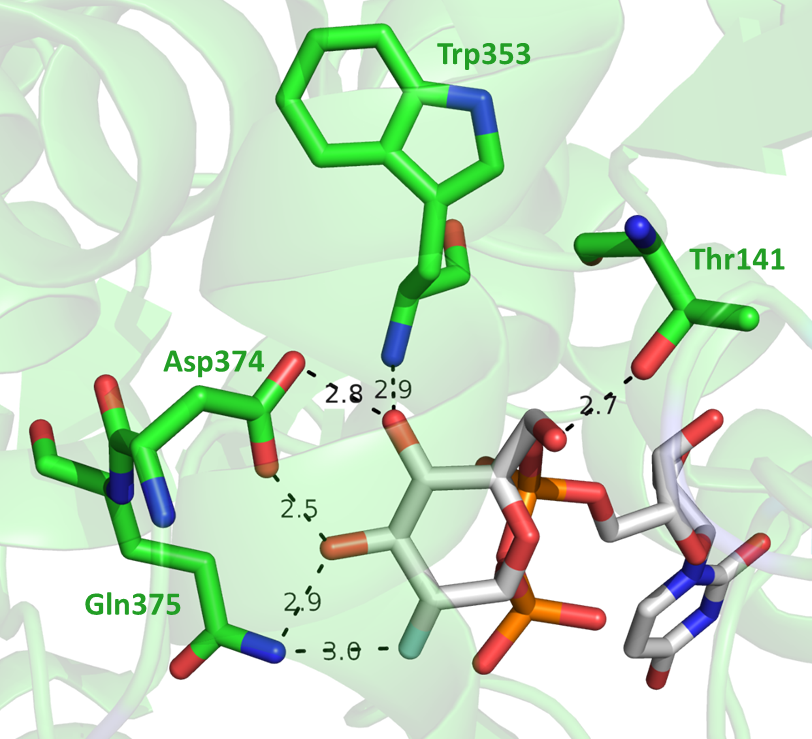


**Supplementary Figure 33**. Active site close-up into the donor binding site of the Michaelis complex of *Vv*GT1^3^ (PDB: 2C1Z) with UDP-2-deoxy-2-fluoro-glucose (gray carbons) showing the conserved residues Gln-Asp-Trp-Thr for the binding of glucose.


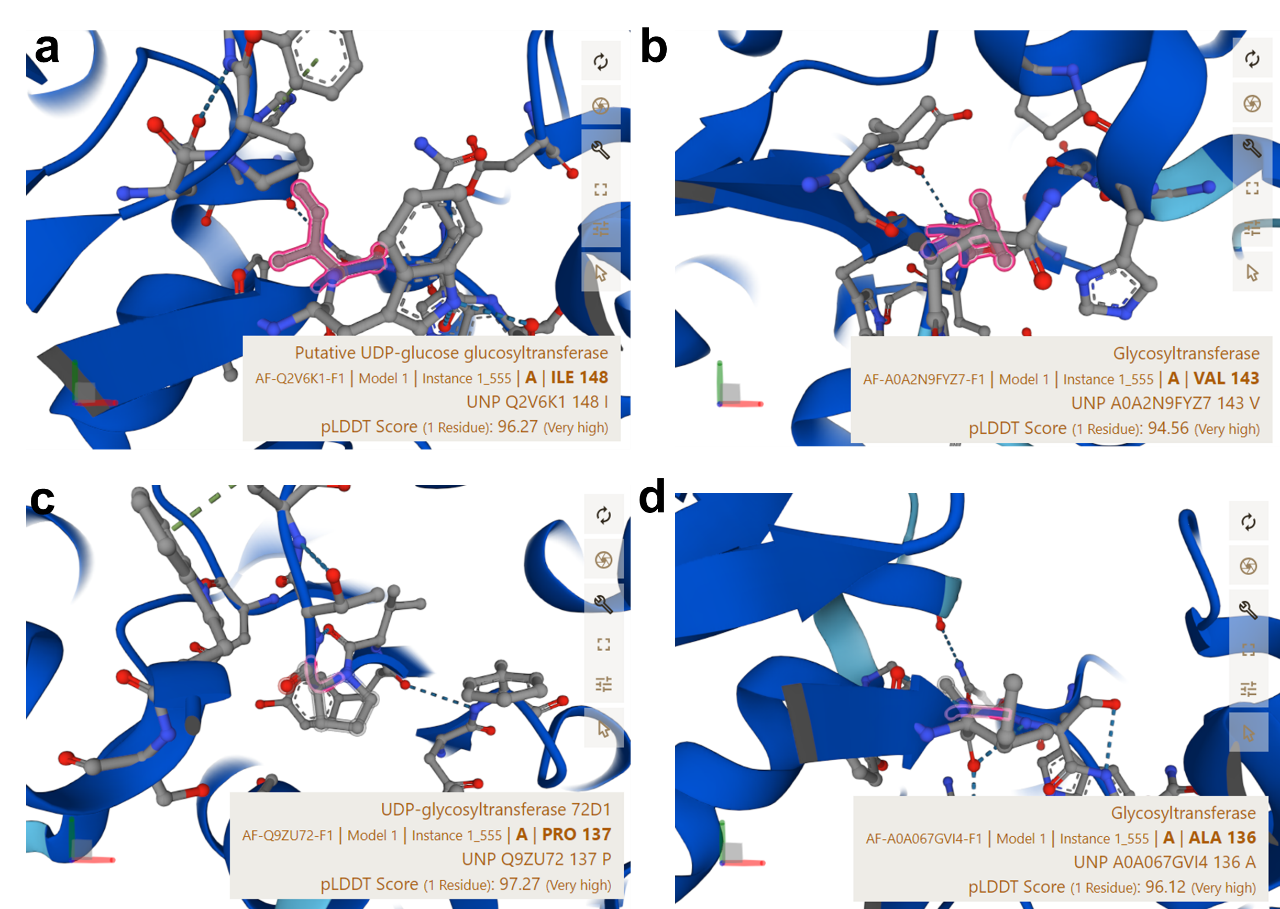


**Supplementary Figure 34**. Structural depiction of pLDDT scores for the residues near glucose C6-OH in the AlphaFold-predicted structures of UGT84A49 (**a**), UGT84A119 (**b**), UGT72D1 (**c**) and UGT72D7 (**d**).


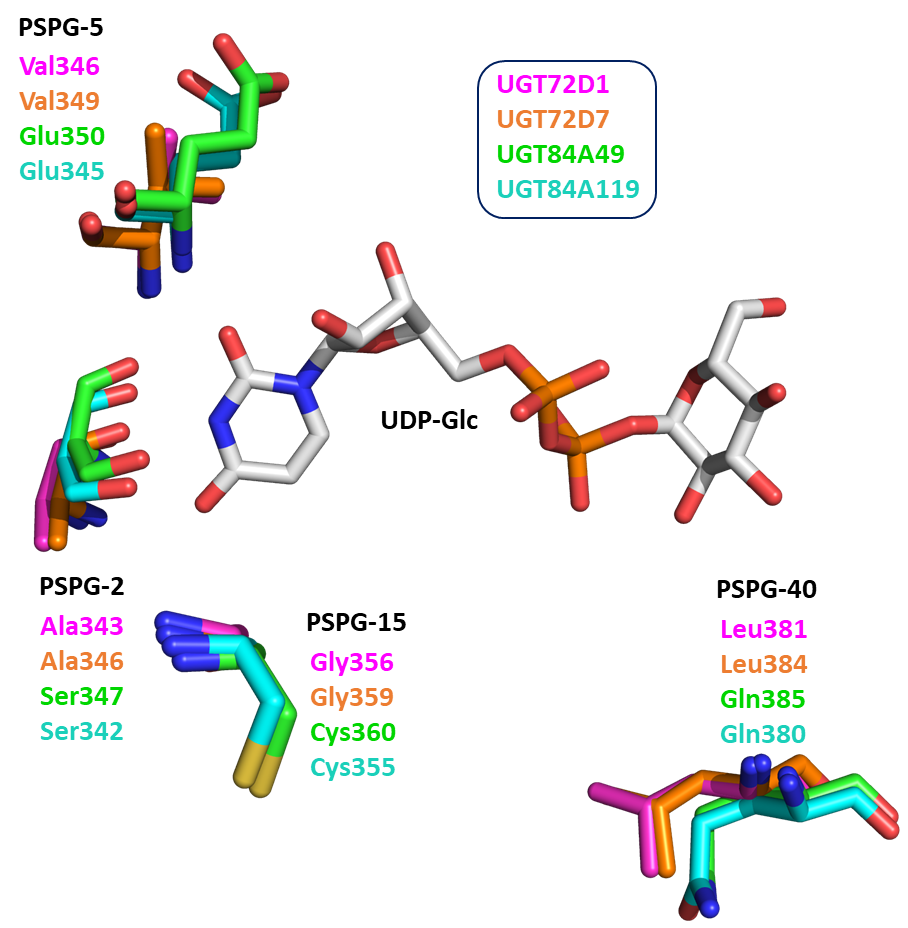


**Supplementary Figure 35**. Active site overlay of the AlphaFold-predicted UGT structures showing the residues in PSPG box positions 2, 5, 15 and 40. UDP-glucose is from the donor complex of *Gg*CGT (PDB: 6L5P^4^).


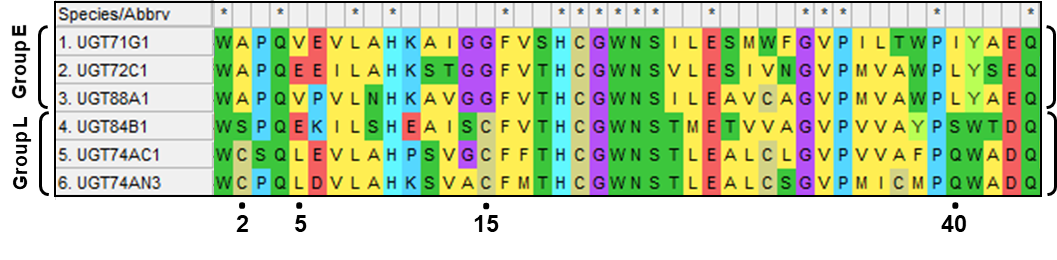


**Supplementary Figure 36**. Multiple sequence alignment of the PSPG motifs of selected UGTs from groups E and L. The PSPG box positions 2, 5, 15 and 40 are highlighted below the alignment.


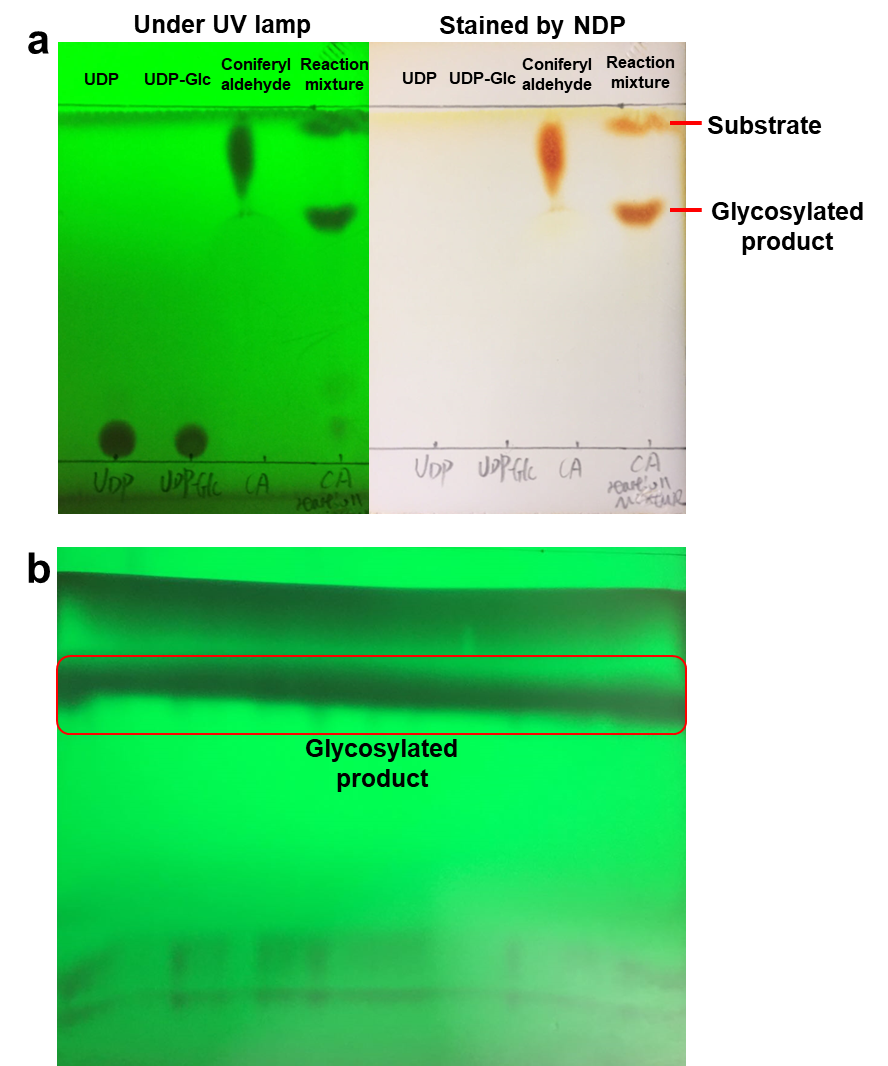


**Supplementary Figure 37**. Analysis and isolation of glycosylated product of coniferyl aldehyde by TLC. **a**. TLC analysis of the UGT72D1/coniferyl aldehyde reaction mixture, visualized under a UV lamp (left) or dyed by dinitrophenylhydrazine (DNP) stain (right). **b**. Silica plate used for the isolation of coniferyl aldehyde 4-*O*-glucoside from preparative UGT72D1 reactions, visualized under a UV lamp. DNP stain could react with aldehydes and form the corresponding hydrazones, which are usually yellow to orange.

**Supplementary References**

1. Savino, S., Borg, A. J.E., Dennig, A. et al. Deciphering the enzymatic mechanism of sugar ring contraction in UDP-apiose biosynthesis. *Nat. Catal.* **2**, 1115-1123 (2019).

2. Nidetzky, B., Gutmann, A., & Zhong, C. Leloir glycosyltransferases as biocatalysts for chemical production. *ACS Catal.* **8**, 6283-6300 (2018).

3. Offen, W. et al. Structure of a flavonoid glucosyltransferase reveals the basis for plant natural product modification. *EMBO J.* **25**, 1396-1405 (2006).

4. Zhang, M. et al. Functional characterization and structural basis of an efficient di-*C*-glycosyltransferase from *Glycyrrhiza glabra*. *J. Am. Chem. Soc.* **142**, 3506-3512 (2020).
